# Mitochondrial Morphology and Fundamental Parameters of the Mitochondrial Respiratory Chain Are Altered in *Caenorhabditis elegans* Strains Deficient in Mitochondrial Dynamics and Homeostasis Processes

**DOI:** 10.1371/journal.pone.0130940

**Published:** 2015-06-24

**Authors:** Anthony L. Luz, John P. Rooney, Laura L. Kubik, Claudia P. Gonzalez, Dong Hoon Song, Joel N. Meyer

**Affiliations:** 1 Nicholas School of the Environment, Duke University, Durham, North Carolina, United States of America; 2 Simulation Group, Samsung SDI, Suwon-si, Gyeonggi-do, Republic of Korea; Universidad Pablo de Olavide, Centro Andaluz de Biología del Desarrollo-CSIC, SPAIN

## Abstract

Mitochondrial dysfunction has been linked to myriad human diseases and toxicant exposures, highlighting the need for assays capable of rapidly assessing mitochondrial health *in vivo*. Here, using the Seahorse XF^e^24 Analyzer and the pharmacological inhibitors dicyclohexylcarbodiimide and oligomycin (ATP-synthase inhibitors), carbonyl cyanide 4-(trifluoromethoxy) phenylhydrazone (mitochondrial uncoupler) and sodium azide (cytochrome c oxidase inhibitor), we measured the fundamental parameters of mitochondrial respiratory chain function: basal oxygen consumption, ATP-linked respiration, maximal respiratory capacity, spare respiratory capacity and proton leak in the model organism *Caenhorhabditis elegans*. Since mutations in mitochondrial homeostasis genes cause mitochondrial dysfunction and have been linked to human disease, we measured mitochondrial respiratory function in mitochondrial fission (*drp-1*)-, fusion (*fzo-1*)-, mitophagy (*pdr-1*, *pink-1*)-, and electron transport chain complex III (*isp-1*)-deficient *C*. *elegans*. All showed altered function, but the nature of the alterations varied between the tested strains. We report increased basal oxygen consumption in *drp-1*; reduced maximal respiration in *drp-1*, *fzo-1*, and *isp-1*; reduced spare respiratory capacity in *drp-1* and *fzo-1*; reduced proton leak in *fzo-1* and *isp-1*; and increased proton leak in *pink-1* nematodes. As mitochondrial morphology can play a role in mitochondrial energetics, we also quantified the mitochondrial aspect ratio for each mutant strain using a novel method, and for the first time report increased aspect ratios in *pdr-1*- and *pink-1*-deficient nematodes.

## Introduction

Mitochondria play many important roles in cellular and organismal health including apoptosis [1], retrograde signaling [2], Ca^2+^ signaling [3], and the Krebs cycle [4]; however, mitochondria are best known for ATP production via oxidative phosphorylation (OXPHOS). The importance of mitochondria in organismal heath is highlighted by the fact that mitochondrial dysfunction is causal in myriad human diseases affecting at least 1 in 5,000 individuals [5], and has been implicated in contributing to many others [6, 7]. Furthermore, many drugs [8] and pollutants [9] cause mitochondrial dysfunction, in some cases only in specific (sensitive) genetic backgrounds [10, 11], thus emphasizing the need for a better understanding of the effects of genetic deficiencies and environmental exposures on mitochondrial health *in vivo*.

Mitochondria are dynamic organelles that respond to cellular and/or environmental cues through fission and fusion. These interlinked processes are critical for maintaining proper mitochondrial function, number and shape [12]. Mitochondrial dynamics are also crucial for stress response, as damaged mitochondria can fuse, allowing contents to mix, and then undergo fission generating two healthy mitochondria in a process termed functional complementation [13]. Damaged mitochondria can also undergo fission, segregating damaged components, which can undergo degradation via targeted autophagy or mitophagy [14], thus preserving a healthy mitochondrial network. Further highlighting the importance of mitochondrial dynamics in organismal health, mutations in human fusion genes *OPA1* and *MFN2* cause dominant optic atrophy [15] and Charcot Marie Tooth Neuropathy type 2A [16], respectively, while mutations in mitophagy genes *PINK1* and *PARK2* cause familial Parkinson’s disease [17], and mutations in the fission gene, *DRP1*, have been associated with rare cases of neurodegeneration and early death [18].

Experiments in whole organisms are important because intercellular signals and cellular context that may affect mitochondrial function can be lost in *in vitro* experiments [19]. *Caenorhabditis elegans* is a free-living nematode found largely in decaying leaf litter [20]. As a model organism *C*. *elegans* offers many advantages over traditional mammalian models, including a short (2–3 week) lifecycle, ease of maintenance, and potential for medium-throughput experiments [21, 22]. Conservation of many molecular and cellular pathways [23], a fully sequenced and annotated genome [24], availability of genetic mutants [25], and ease of genetic knockdown via RNA interference [26, 27] contribute further to the utility of *C*. *elegans* as a model for studying mitochondrial dysfunction *in vivo*. Tools for assessment of mitochondrial function in *C*. *elegans* currently include time-consuming biochemical analysis of extracts [28], *in vivo* analysis of ATP levels using a transgenic reporter [29], and analysis of oxygen consumption (basal respiration) using individual or multiwell plate formats [30].

Here we describe how to assay the fundamental parameters of mitochondrial respiratory chain function, including basal oxygen consumption rate (OCR), maximal respiratory capacity, spare respiratory capacity, ATP coupled respiration, and proton leak with pharmacological inhibitors of the electron transport chain (ETC) in the model organism *C*. *elegans* using the Seahorse XF^e^24 Analyzer (Seahorse Bioscience, Massachusetts, USA). Furthermore, we report alterations in these parameters in nematodes carrying mutations in orthologs of the human outer membrane fusion gene *MFN2*, mitochondrial fission gene *DRP1*, mitophagy genes *PINK1* and *PARK2*, and a complex III Rieske iron sulfur protein (*fzo1*, *drp-1*, *pink-1*, *pdr-*1 and *isp-1*, respectively, in *C*. *elegans*). These results highlight the importance of mitochondrial dynamics in maintaining proper mitochondrial function. Clearly, however, the analysis of mitochondrial function in nematodes carrying mutations in genes of other critical mitochondrial pathways, such as apoptosis, the ETC, the Krebs cycle or fatty acid oxidation will help us better understand connections between these pathways and mitochondrial energetics.

## Materials and Methods

### *C*. *elegans* Culture

Bristol N2 (wild-type), MQ887 *isp-1* (*qm150;* outcrossed 3x), VC1024 *pdr-1* (*gk448*; outcrossed 3x), and CB6193 *bus-8* (*e2885;* outcrossed 3x) *C*. *elegans* were purchased from the Caenorhabditis Genetics Center (CGC, University of Minnesota). CU5991 *fzo-1* (*tm1133*; outcrossed 4x) were provided by Alexander van der Bliek, University of California (Los Angeles, CA, USA), *pink-1* (*tm1779*; outcrossed 1x) were provided by Guy Caldwell, University of Alabama, and CU6372 *drp-1* (*tm1108;* outcrossed 9x) were provided by Ding Xue, University of Colorado. All mutant strains will henceforth be referred to by their gene name. Synchronized populations of *C*. *elegans* were obtained by sodium hydroxide bleach treatment as previously described [31], followed by overnight incubation in complete K-medium on a shaker at 20C [32]. Age synchronized L1 (larval stage one) nematodes were then maintained at 20C on K-agar plates [33] seeded with OP50 *Escherichia coli* until L4 (larval stage four) was reached (approximately 48 hours for N2, *drp-1*, *pdr-1*, *pink-1* and 72 or 96 hours for slow growing *fzo-1* and *isp-1*, respectively).

### Drug Preparation

Dicyclohexylcarbodiimide (DCCD), oligomycin A, carbonyl cyanide 4-(trifluoromethoxy) phenylhydrazone (FCCP), and 2,4-dinitrophenol (Sigma Chemical Co., St. Louis, MO) stocks were prepared in dimethyl sulfoxide (DMSO), and diluted in unbuffered reconstituted hard water (“EPA water” hereafter) (60mg MgSO_4_·7H_2_O, 60mg CaSO_4_·2H_2_O, 4mg KCl per liter ddH_2_O) [34] to their final working concentrations. Sodium azide (Sigma Chemical Co., St. Louis, MO) was dissolved in unbuffered EPA water to a final working stock of 80mM.

### Sample Preparation

All experiments were performed with synchronized L4 *C*. *elegans*, as the L3/L4 transition is accompanied by a dramatic increase in mtDNA copy number and demand for oxidative phosphorylation [35–38]. L4 *C*. *elegans* were rinsed from OP50 K-agar plates into sterile 15mL centrifuge tubes, washed twice with K-medium, and allowed to clear their guts for 20 minutes to remove contaminating bacteria that might otherwise confound oxygen consumption rate (OCR) measurements. Next, nematodes were resuspended in unbuffered EPA water to an approximate concentration of one worm per microliter (estimated by counting the number of worms in 20μl drops)**.** Approximately 75 nematodes were then pipetted into each well of a 24-well Seahorse utility plate using tips rinsed in 0.1% Triton X-100 to prevent worm loss due to sticking. The final volume of each well was then brought to 525μl with unbuffered EPA water. At least two wells per assay were left as blanks. 75μL of 160μM DCCD (8% DMSO), 120μM FCCP (16% DMSO), and 80mM azide were then pipetted into the appropriate injection ports of the seahorse cartridge. After injection, each drug solution is diluted by a factor of eight to the appropriate final concentrations (i.e. 20μM DCCD (1% DMSO), 15uM FCCP (2% DMSO), and 10mM azide).

Seahorse programs were set up such that each oxygen consumption measurement consisted of a one minute mix cycle (which oxygenates the micro-chamber), followed by a three minute wait period (to allow worms to settle), and finally a three minute interval for measurement of oxygen levels. Eight oxygen consumption measurements were taken for determination of basal OCR. Drugs were then injected, and fourteen, eight or four OCR measurements were taken at eight minute intervals after DCCD, oligomycin, FCCP, or azide injections, respectively. Over the course of the assay it is important to monitor oxygen levels per well, as exposing the nematodes to hypoxic conditions could confound the measurements; oxygen levels below 100 mmHg were taken as an indicator of excess number of nematodes per well (Kevin Bittman, Ph.D., Field Applications Scientist, Seahorse Biosciences, Inc., personal communication). It is also critical to note whether or not the mix cycle is fully re-oxygenating the micro-chamber, and to readjust the mix cycle as needed.

Basal OCR measurements were highly variable over the initial four readings, but then stabilized. Therefore, we averaged the final four measurements to obtain an average basal OCR per well. The nematodes’ response to DCCD was not instantaneous, but OCR measurements consistently decreased, and plateaued between the sixth and eighth measurements. Therefore, we averaged the final six measurements to obtain our OCR in response to DCCD. *C*. *elegans’* response to FCCP, although not instantaneous, was much more rapid than for DCCD. Thus, we averaged the final six measurements to obtain the average OCR in response to FCCP. Response to azide was essentially instantaneous, so we averaged all four measurements. Finally, we normalized all data to both worm number and total protein as measured by BCA assay (Thermo Fisher Scientific, Rockford, IL). Using this method we treated each individual well as an “n” of one. All experiments were run at least two to three times, separated in time.

Typically, when using a Seahorse instrument with cells in culture, inhibitors are injected into each well of a seahorse utility plate in tandem, such that after basal OCR is measured the ATP synthase inhibitor is injected (DCCD or oligomycin), followed by the mitochondrial uncoupler (2,4-DNP or FCCP), and finally by a complete respiratory inhibitor (sodium azide or rotenone plus antimycin A). This strategy allows for the determination of basal OCR, maximal respiratory capacity, spare respiratory capacity, proton leak, and ATP turnover for each well. However, this strategy does not appear to be possible in *C*. *elegans*. When we injected the cytochrome c oxidase inhibitor sodium azide after the final FCCP measurement, we found that the magnitude of the nematode’s response to sodium azide was diminished (S1 Fig, one way ANOVA, main effect of treatment P < .0001). To avoid this problem, sequential injection of other complete respiratory inhibitors such as cyanide or rotenone and antimycin A may be possible. In the experiments reported here, each drug was injected into a separate well to obtain reliable, reproducible results.

### Extracellular Acidification Rate (ECAR)

Due to the dual probe capacity of the Seahorse XF^e^24 it is possible to obtain oxygen consumption and extracellular acidification rate (ECAR) simultaneously, which is why all assays were run in unbuffered EPA water. Simultaneous measurements of OCR and ECAR have proven valuable in the context of toxicant exposures that can cause a shift in metabolism from OXPHOS to aerobic glycolysis [39], otherwise known as the Warburg effect [40]. To test whether we could measure ECAR in *C*. *elegans*, we tested ECAR in wild-type and *fzo-1* nematodes. *fzo-1 (tm1133)* nematodes, in which mitochondria are highly fragmented and exhibit intracellular acidification, likely due to increased glycolysis, because sodium dichloracetate (a pyruvate dehydrogenase stimulator) alleviates acidosis [41]. However, no differences in ECAR were noted between L4 stage N2 and *fzo-1* nematodes (S2 Fig, one way ANOVA, P>0.05). We speculate that extrusion of glycolytic byproducts in *C*. *elegans* (such as lactate) does not occur in the same manner as cells in culture, thus limiting the value of ECAR measurements.

### Mitochondrial Morphology

*C*. *elegans* were picked onto agar plates seeded with either OP50 (negative control) or OP50 containing 3.7μM MitoTracker Red CMXROS (Molecular Probes, Invitrogen) and incubated overnight. Worms incubated with MitoTracker were picked onto plates seeded with OP50 for 30 minutes the next day to allow the dye to clear out of the gut. Each strain was then picked onto an agar pad containing levamisole (25mg/mL) and subsequently imaged on a confocal microscope (Zeiss 510 upright, Duke Light Microscopy Core Facility).

Raw images were converted to binary images using MATLAB. First, maximum projection technique as described in [42] was applied in order to combine z-stack images into a single image. Gray values of this image were linearly transformed to cover the entire 16 bit gray scale. These images were then deblurred using the ‘deconvblind’ function. The experimentally measured point spread function (PSF) was applied as an initial PSF for this deconvolution process. A Gaussian low pass filter was applied to smooth the edges of the boundary. Finally, these images were converted to the binary images using Otsu’s method [43].

**Fig 1:**
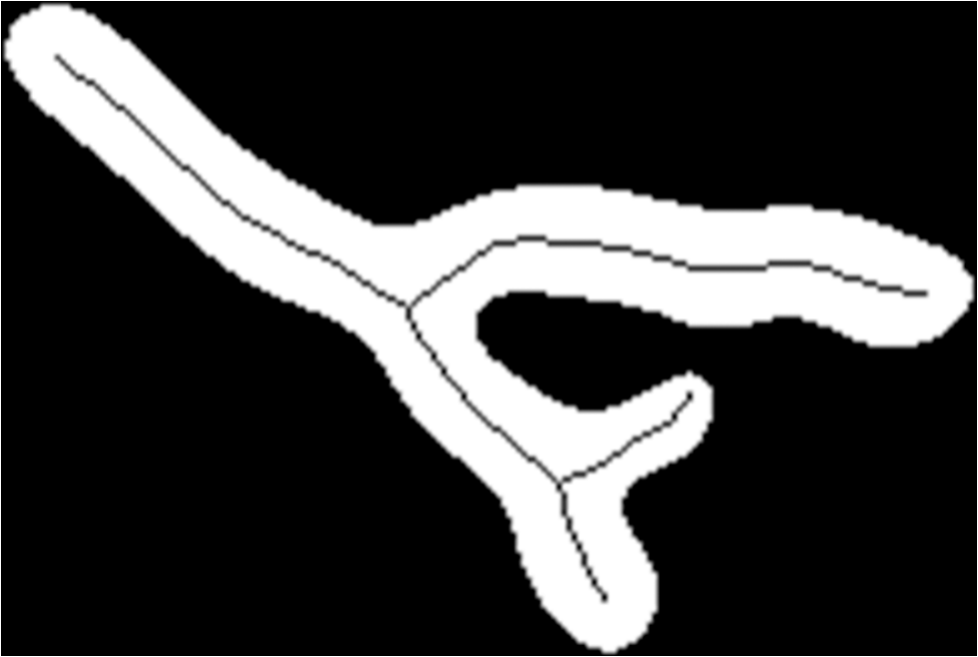
Image processing result of a branched mitochondrion. A centerline was extracted using a morphological thinning operation.

As can be seen in Fig 1, the centerline of a mitochondrion was extracted from the binary image using a morphological thinning operation in MATLAB (‘bwmorph’ function with ‘thin’ operation). After the thinning operation, the endpoints of the centerline were connected to the boundary of a mitochondrion to complete the centerline. The average width of a mitochondrion was then calculated as area divided by the centerline length. The aspect ratio (AR) was defined as the ratio between centerline length and average width (multiplying by π/4 allows the aspect ratio of a circle to be one).

$$\text{A R}=\frac{\pi }{4}\frac{\text{centerline length}}{\text{average width}}$$

As we observed some nonspecific dye uptake in the gut, we measured mitochondria from lateral body wall muscle cells from three worms within a strain (n = 108–173). Our analysis focused on strain to strain differences and pair wise comparisons to wild type nematodes were performed for each strain using a one way ANOVA.

### Statistics

All statistics were performed using JMP v11.0 software (SAS Institute). All OCR data was initially assessed with a one or two way ANOVA. When appropriate, post-hoc analysis was carried out using a Student’s t-test.

## Results

### Drug Titrations

We first carried out titrations of inhibitors of various ETC components to allow us to measure different aspects of mitochondrial function.

#### Oligomycin and Dicyclohexylcarbodiimide

Oligomycin and DCCD are ATP synthase inhibitors that bind the F_O_ and F_O_F_1_ subunits of the complex, respectively, preventing proton translocation and phosphorylation of ADP to ATP [44]. Inhibition of ATP synthase provides a measure of the amount of oxygen consumption coupled directly to ATP production. However, in N2 nematodes, oligomycin proved ineffective at inhibiting ATP synthase at all concentrations tested (5–50μM, 2% DMSO), likely due to limited penetration of the nematode’s collagenous cuticle by this bulky compound [45] over the timeframe of the assay (up to 15 cycles tested, or roughly 1.75 hours) (S3 Fig). Higher concentrations of oligomycin could not be tested, due to its limited water solubility. Citreoviridin A, another ATP synthase inhibitor [46, 47], was also tested, but to no effect.

**Fig 2:**
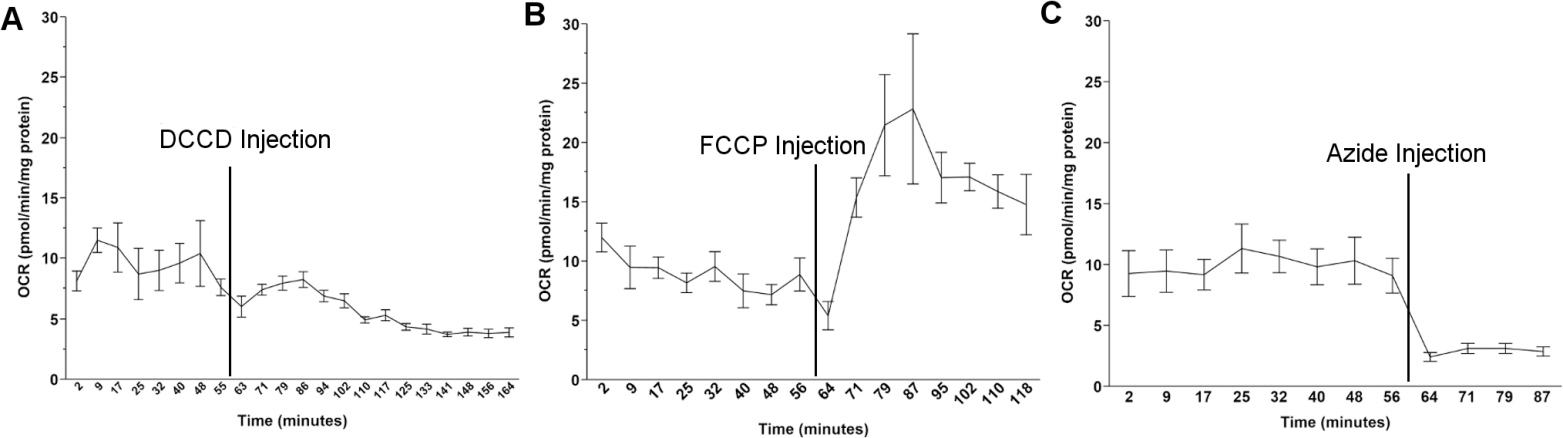
Representative Seahorse XFe24 output data for L4 N2 nematodes dosed with (A) DCCD, (B) FCCP, and (C) sodium azide.

DCCD inhibited ATP synthase more effectively than either oligomycin or citreoviridin. DCCD had a significant effect on OCR (one way ANOVA, main effect of treatment P<0.0001). 10 and 20μM DCCD significantly reduced OCR from basal levels (P<0.0001 for both pairwise comparisons), while 5 and 50μM DCCD did not alter OCR (P = 0.2 and 0.3, respectively, for pairwise comparisons). DCCD is a water insoluble compound and it is plausible that DCCD precipitated out of solution over the time course of the assay, explaining why 50μM DCCD failed to effect OCR. However, our 2% DMSO control caused a slight but significant increase in OCR rates (P = 0.004 for pairwise comparison) (S4 Fig). To avoid this confounding DMSO effect, we reduced our final DMSO concentrations to 1%, the lowest possible DMSO concentration where 20μM DCCD, the most effective concentration tested, was soluble. DCCD significantly reduced OCR at all concentrations of DMSO tested (one way ANOVA, main effect of treatment, P<0.0001). 20μM DCCD at 1, 1.5, and 2% final DMSO concentrations all significantly reduced OCR (P<0.05 for all pairwise comparisons), while DMSO concentration did not affect the efficacy of 20μM DCCD in reducing OCR (P>0.05 for all pairwise comparisons) (S5 Fig). Since 1% DMSO did not significantly affect basal OCR (P = 0.5 for pairwise comparison), we chose to use µM DCCD at a final DMSO concentration of 1% for all future experiments. Representative Seahorse XF^e^24 output data for 20μM DCCD (1% DMSO) is shown in Fig 2A.

Although DCCD and oligomycin are both ATP synthase inhibitors, DCCD lacks the specificity of oligomycin and is capable of inhibiting other cellular ATPases; thus, it gives an imperfect measure of ATP-linked respiration and proton leak [44]. We explored two alternatives that might permit the use of oligomycin instead of DCCD. First, we attempted long-term (12H) pre-incubation with oligomycin and second, we titrated DCCD and oligomycin in cuticle-deficient *bus-8* nematodes that have also been reported to be hypersensitive to several bulky drugs [48]. A 12 hour pre-incubation with oligomycin resulted in a significant reduction in basal OCR in *bus-8* nematodes (one way ANOVA, main effect of treatment P<0.0001). 10, 25 and 50μM oligomycin significantly reduced basal OCR (P = 0.004, P<0.0001, P<0.0001, respectively, for all pairwise comparisons), with 50μM causing roughly a 40% reduction in OCR (S18 Fig). When injected in real-time, both oligomycin and DCCD inhibited ATP synthase in *bus-8* nematodes and caused significant reductions in OCR (one way ANOVA, main effect of treatment P = 0.0007 and P<0.0001, respectively). 25 and 50μM oligomycin caused significant reductions in OCR (P = 0.0017 and P = 0.005, respectively, for pairwise comparisons), whereas 10μM had no effect (P>0.05) (S19 Fig). 5, 10 and 20μM DCCD caused significant reductions in OCR (P<0.0001 for all pairwise comparisons) and 1μM had no effect (P>0.05) (S20 Fig). The magnitude of the effect for the two drugs were significantly different when we compared the percent reduction in basal OCR after administration of 20μM DCCD or 50μM oligomycin (one way ANOVA, P = 0.0005) (S21 Fig), with DCCD causing roughly a 55–60% decrease and oligomycin causing a 35–40% decrease.

#### Carbonyl cyanide 4-(trifluoromethoxy) phenylhydrazone and 2,4-Dinitrophenol

FCCP and DNP are potent uncouplers of mitochondrial oxidative phosphorylation [49, 50] that dissipate the proton gradient in the mitochondrial intermembrane space by transporting protons across the inner mitochondrial membrane independently of ATP synthase activity. Once in the mitochondrial matrix, uncouplers deprotonate, cross back into the intermembrane space, and repeat the cycle, thus uncoupling oxygen consumption from ATP production (oxygen is still consumed to generate and maintain the proton gradient, but ATP is not produced). Dissipation of the proton gradient forces increased fuel oxidation, oxygen consumption, and thus proton pumping in an attempt to re-establish the proton gradient. Therefore, uncoupling provides a useful measure of maximal respiratory capacity, or an organism’s ability to respond to increasing energy demands [51].

2,4-DNP (25–100μM, 2% DMSO) had no effect on mitochondrial respiration over the time course tested (S6 Fig), while a near instantaneous increase in OCR was observed after FCCP treatment. Other groups have also reported a lack of mitochondrial uncoupling after 2,4-DNP treatment in nematodes [52–54]; however the exact reason for this is unknown. One possible explanation for the lack of effect of 2,4-DNP is that it is highly ionized (776:1, based on a pK_a_ of 4.11) when injected in EPA water (pH~7), whereas FCCP is less so (16:1, based on a pK_a_ of 5.8), and it is likely the ionized compounds do not penetrate the nematode cuticle efficiently. Thus titration experiments were performed to identify the appropriate FCCP concentration for effective mitochondrial uncoupling.

FCCP treatment had a significant effect on OCR (one way ANOVA, P<0.0001). 5, 15, 25 and 50μM FCCP (2% DMSO) significantly increased OCR measurements above basal rates (P<0.0001 for all pairwise comparisons to control), while our 2% DMSO control did not significantly increase OCR (P = 0.3 for pairwise comparison) (S7 Fig). The fact that our 2% DMSO control failed to significantly increase OCR in our FCCP trials despite increasing OCR after DCCD injections is likely due to the different lengths of the assays, as we measure OCR 14 times post-DCCD injection, while only eight times post-FCCP. In contrast to DCCD, titrating our final DMSO concentrations downward resulted in increased variability in the FCCP response (S8 Fig) so we chose to run all future experiments with 2% DMSO. Since 15, 25, and 50μM FCCP did not elicit statistically significantly different responses (P>0.05 for all pairwise comparisons), we ran all future experiments with the lowest concentration of FCCP, 15μM, which is similar to concentrations other groups have used to uncouple respiration in nematodes [55]. Representative Seahorse XF^e^24 output data for 15μM FCCP (2% DMSO) is shown in Fig 2B.

#### Sodium Azide

Azide is a powerful inhibitor of mitochondrial respiration, and works by inhibiting complex IV (cytochrome c oxidase (COX)) of the ETC by binding directly to the heme prosthetic group, preventing the final transfer of electrons to oxygen [56, 57]. The selectivity of azide as a COX inhibitor has been questioned, as early *in vitro* studies suggested that azide promiscuously inhibits myriad heme containing enzymes. However, azide binding is pH dependent due to its pK_a_ (4.7), and at physiological pHs azide preferentially exists as the free anion (N_3_^-^). Most heme containing enzymes preferentially bind the protonated form of azide, but COX is an exception, binding the anion with greater affinity [58]. This suggests that azide has high specificity for COX inhibition *in vivo*, making it an ideal drug for our studies.

Treatment with sodium azide had a significant effect on nematode OCR (one way ANOVA, P<0.0001). 2.5, 5, 10, and 15mM azide all significantly reduced OCR below basal levels (P<0.0001 for all pairwise comparisons to control), and the azide response was not statistically different for any of the azide concentrations tested (P>0.05 for all pairwise comparisons) (S9 Fig). All future experiments were run with a final concentration of 10mM. Representative Seahorse XF^e^24 output data for 10mM azide is shown in Fig 2C.

### Mutant *C*. *elegans* Metabolic Profiles and Mitochondrial Morphologies

Having identified appropriate conditions for analysis of mitochondrial function, we next carried out analyses of mitochondrial function in strains of *C*. *elegans* carrying mutations in critical mitochondrial function and homeostasis genes. Because some of the strains we used grow to different sizes, we report our results on both a per unit protein and per individual nematode basis. Alterations in mitochondrial morphology for many, but not all of these mutants have been previously reported; we carried out further morphological analysis using a dye-based imaging technique and novel imaging method that complements previous literature. All results are summarized in Table 1.

**Table 1:** Summary of alterations in fundamental parameters of the mitochondrial respiratory chain in nematodes deficient in mitochondrial dynamics and homeostasis processes.

Mitochondrial Parameter*						
Strain	Aspect Ratio	Basal OCR (pmol/min/mg protein; P<0.0001)	ATP Linked OCR (pmol/min/mg protein; P = 0.65)	Maximal OCR (pmol/min/mg protein; P<0.0001)	Spare Capacity (pmol/min/mg protein; P = 0.022)	Proton Leak (pmol/min/mg protein; P<0.0001)
N2	4.24±0.29 (n = 147)	8.78±0.34 (n = 37)	4.27±0.44 (n = 12)	18.40±0.74 (n = 13)	8.51±0.96 (n = 13)	1.15±0.33 (n = 8)
CU5991 *fzo-1* (*tm1133*)	1.78±0.06 (n = 131; **P<0.0001**)	8.74±0.28 (n = 55; P = 0.816)	6.24±0.50 (n = 15)	13.40±0.72 (n = 21; **P<0.0001**)	4.64±0.91 (n = 21; **P = 0.020**)	0.34±0.18 (n = 10; **P = 0.039**)
CU6372 *drp-1* (*tm1108*)	4.50±0.40 (n = 108; P = 0.593)	11.3±0.72 (n = 52; **P = 0.0006**)	3.98±0.95 (n = 12)	15.20±1.01 (n = 25; **P = 0.010**)	4.50±1.30 (n = 21; **P = 0.013**)	1.83±0.20 (n = 10; P = 0.085)
*pink-1* (*tm1779*)	5.36±0.35 (n = 173; **P = 0.016**)	9.47±0.46 (n = 35; P = 0.452)	4.16±0.76 (n = 12)	19.1±1.99 (n = 8; P = 0.680)	8.21±1.23 (n = 8; P = 0.884)	-0.02±0.23 (n = 10; **P = 0.004**)
VC1024 *pdr-1* (*gk448*)	5.19±0.36 (n = 125; **P = 0.032**)	10.20±0.55 (n = 35; P = 0.089)	4.13±0.37 (n = 12)	19.3±2.94 (n = 8; P = 0.560)	9.13±2.56 (n = 8; P = 0.766)	1.78±0.31 (n = 10; P = 0.106)
MQ887 *isp-1* (*qm150*)	NA**	8.29±0.38 (n = 49; P = 0.383)	5.06±0.59 (n = 15)	15.40±0.75 (n = 16; **P = 0.030**)	6.75±0.71 (n = 16; P = 0.309)	2.32±0.32 (n = 9; **P = 0.005**)

*Values are shown as mean ± SE. ANOVA p-values are shown in parentheses under each column heading. N and P-values for post-hoc comparison to N2 nematodes in parentheses (when appropriate).

**isp-1 mitochondria could not be quantified, likely due to poor MitoTracker Red CMXROS uptake due to decreased mitochondrial membrane potential [59, 60].

#### Basal Oxygen Consumption Rate

First, we investigated whether *C*. *elegans* deficient in mitochondrial fission (*drp-1*), fusion (*fzo-1*), mitophagy (*pdr-1*, *pink-1*) or ETC complex III (*isp-1*) activity have altered basal oxygen consumption. Since mitochondrial dynamics are critical in maintaining healthy mitochondrial networks [12], we hypothesized that disruption of these genes would result in altered patterns of oxygen consumption.

**Fig 3:**
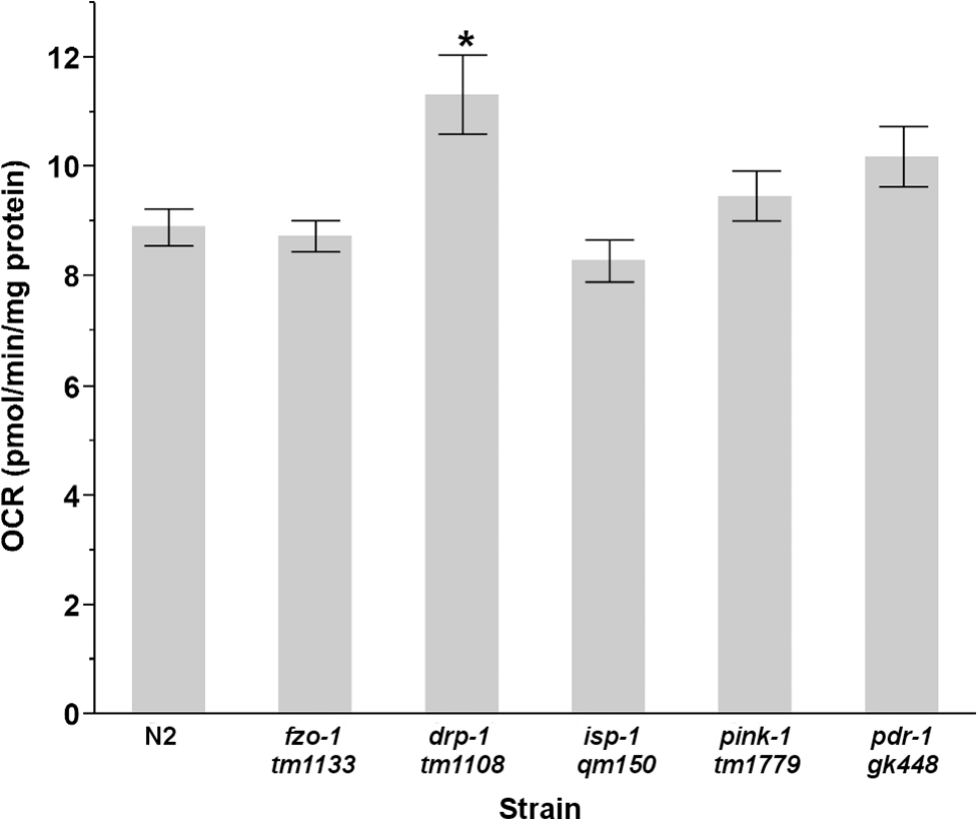
Basal OCR is elevated in L4 *drp-1* nematodes. Statistical significance was analyzed via a one way ANOVA (main effect of strain, P<0.0001) (n = 31–45). Asterisks (*) denote statistical significance. Bars ± SEM.

Basal oxygen consumption was measured using 75 L4 N2, *drp-1*, *pdr-1* and *pink-1* nematodes. 150 L4 *fzo-1* and *isp-1* nematodes were required to meet the Seahorse XF^e^24’s range of detection (40–1400 pmols/min), presumably because L4 *fzo-1* and *isp-1* are smaller than wild-type nematodes. Basal oxygen consumption per unit protein was significantly different between strains (one way ANOVA, main effect of strain, P < .0001) (Fig 3). *drp-1* nematodes had significantly elevated oxygen consumption compared to wild-type (N2) nematodes (P = .0006 for pairwise comparison), which is in agreement with the idea that highly fused mitochondrial networks are more metabolically active [61]. Interestingly, we did not observe decreased oxygen consumption in *fzo-1* or *isp-1* nematodes, which have a missense mutation in the Rieske iron sulfur protein subunit of complex III. However, we did observe a dramatically decreased OCR in *isp-1* on a per-nematode basis, likely due to *isp-1*’s reduced size. OCR in *drp-1* nematodes was also elevated on a per nematode basis (one way ANOVA, P<0.0001, P<0.05 for both pairwise comparisons to control) (S10 Fig).

#### ATP Coupled Respiration

Inhibition of ATP synthase by DCCD provides a measure of the amount of oxygen consumption coupled to ATP production. We hypothesized that mutations in mitochondrial dynamics genes and/or complex III of the ETC, which induce mitochondrial dysfunction, would alter this parameter.

Treatment of N2, *drp-1*, *pdr-1*, *pink-1*, *fzo-1*, and *isp-1* with 20μM DCCD caused a significant reduction in OCR in all strains (S11A Fig) (two-way ANOVA, main effect of strain and treatment, P<0.0001 for both, but not their interaction, P = 0.65). Nematode response to DCCD and ATP-coupled respiration on a per worm basis is shown in S12 Fig, and was similar to the results obtained on a per protein basis.

#### Maximal Respiratory and Spare Respiratory Capacity

**Fig 4:**
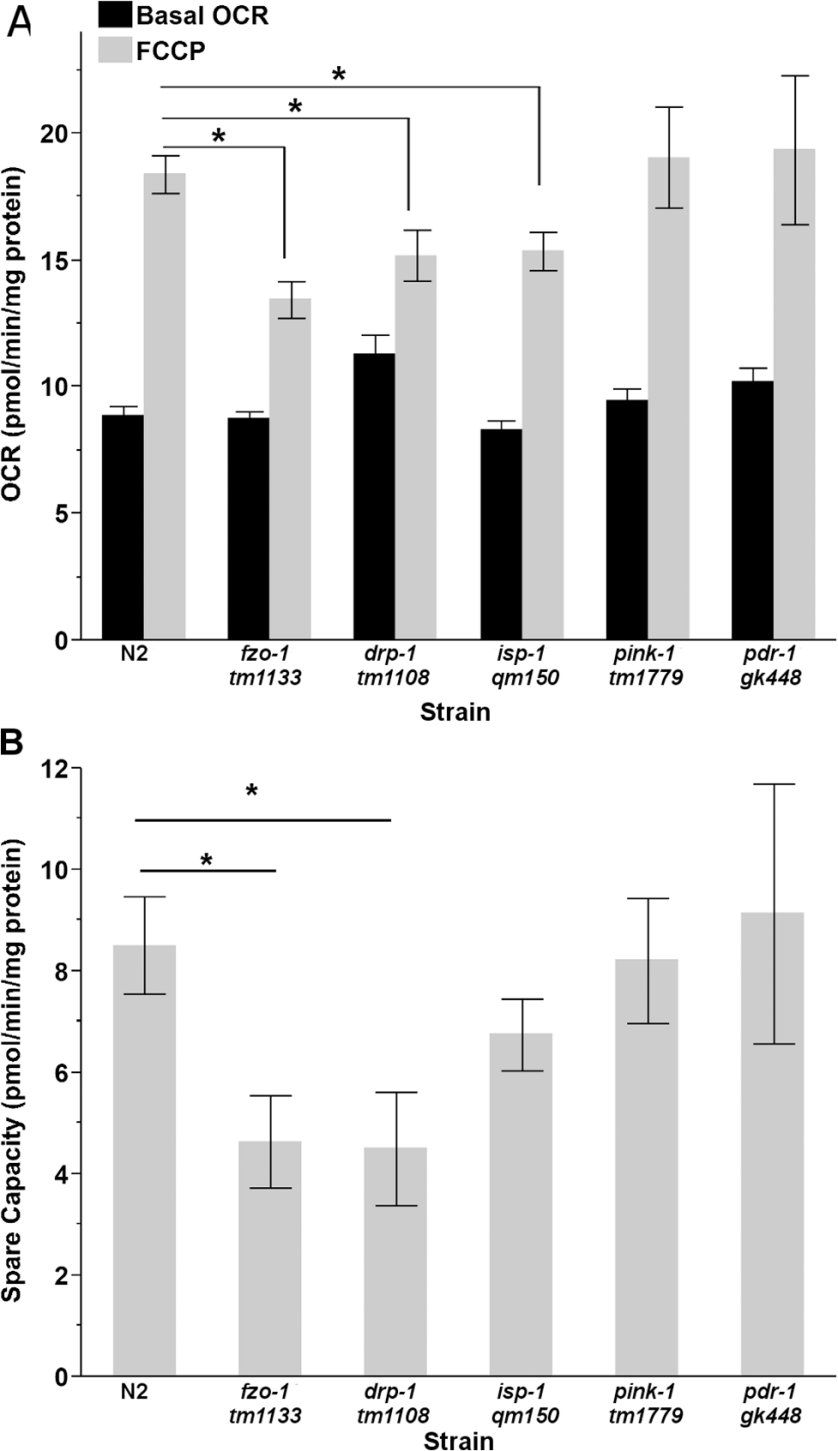
Maximal and spare respiratory capacity in L4 nematodes. (A) Treatment with FCCP caused a significant increase in OCR in all strains (two way ANOVA, main effects of strain, treatment and their interaction, P<0.0001 for all); however, L4 *fzo-1*, *drp-1* and *isp-1* had reduced maximal respiratory capacity compared to N2 nematodes (Student’s t-test, p = 0.03, p<0.0001, p = 0.01, respectively). (B) Spare respiratory capacity was reduced in *fzo-1* and *drp-1*, compared to wild-type nematodes (one way ANOVA, P = 0.022). (n = 12–20). Asterisks (*) denote statistical significance. Bars ± SEM.

Uncoupling ATP production from oxygen consumption with FCCP provides a measure of maximal respiratory capacity. When basal OCR is subtracted from maximal OCR, the result is spare respiratory capacity, an important measure of an organism’s ability to respond to increasing energy demands. Treatment of N2, *drp-1*, *pdr-1*, *pink-1*, *fzo-1*, and *isp-1* with 15μM FCCP caused a significant increase in OCR above basal levels in all strains (two-way ANOVA, main effects of strain (P < .0001), treatment (P < .0001) and their interaction (P < .0001)) (Fig 4A). *isp-1*, *fzo-1*, and *drp-1* had significantly reduced FCCP responses compared to wild-type nematodes (p = 0.03, p<0.0001, p = 0.01, respectively, for pairwise comparisons) (Fig 4A).

Strain-specific differences were observed when spare respiratory capacity was compared (one way ANOVA for effect of strain, p = 0.022). Post-hoc analysis showed that *drp-1* and *fzo-1* have reduced spare respiratory capacities compared to wild-type (N2) nematodes (P = 0.013, P = 0.02, respectively, for pairwise comparisons) (Fig 4B). Maximal and spare respiratory capacity are shown on a per worm basis in S13 Fig, and were similar to the results obtained on a protein-normalized basis, except for lower spare capacity in *isp-1* nematodes. 2% DMSO controls were run in conjunction with FCCP for each strain; however, DMSO had no significant effect on OCR when normalized to total protein (S15 Fig, two way ANOVA, P>0.05) or nematode (S16 Fig, two way ANOVA, P>0.05).

#### Proton Leak

Proton leak is defined as dissipation of the proton gradient across the inner mitochondrial membrane that is not attributable to ATP synthase activity. Basal proton leak mainly occurs via the adenine nucleotide translocase (ANT), which is not regulated, but can differ between cell types [62]. Inducible proton leak is regulated, and is mainly mediated by uncoupling proteins (UCP), which are inducible by fatty acids, superoxide, and by-products of lipid peroxidation [62]. The exact role of inducible proton leak outside of generation of heat is not fully understood; however, one hypothesis is that proton leak serves a role in a negative feedback loop to limit superoxide production [63]. Mitochondrial dysfunction, caused by mutations in mitochondrial dynamics and ETC complex III, might result in altered levels of proton leak either directly or indirectly, e.g. via compensatory responses.

**Fig 5:**
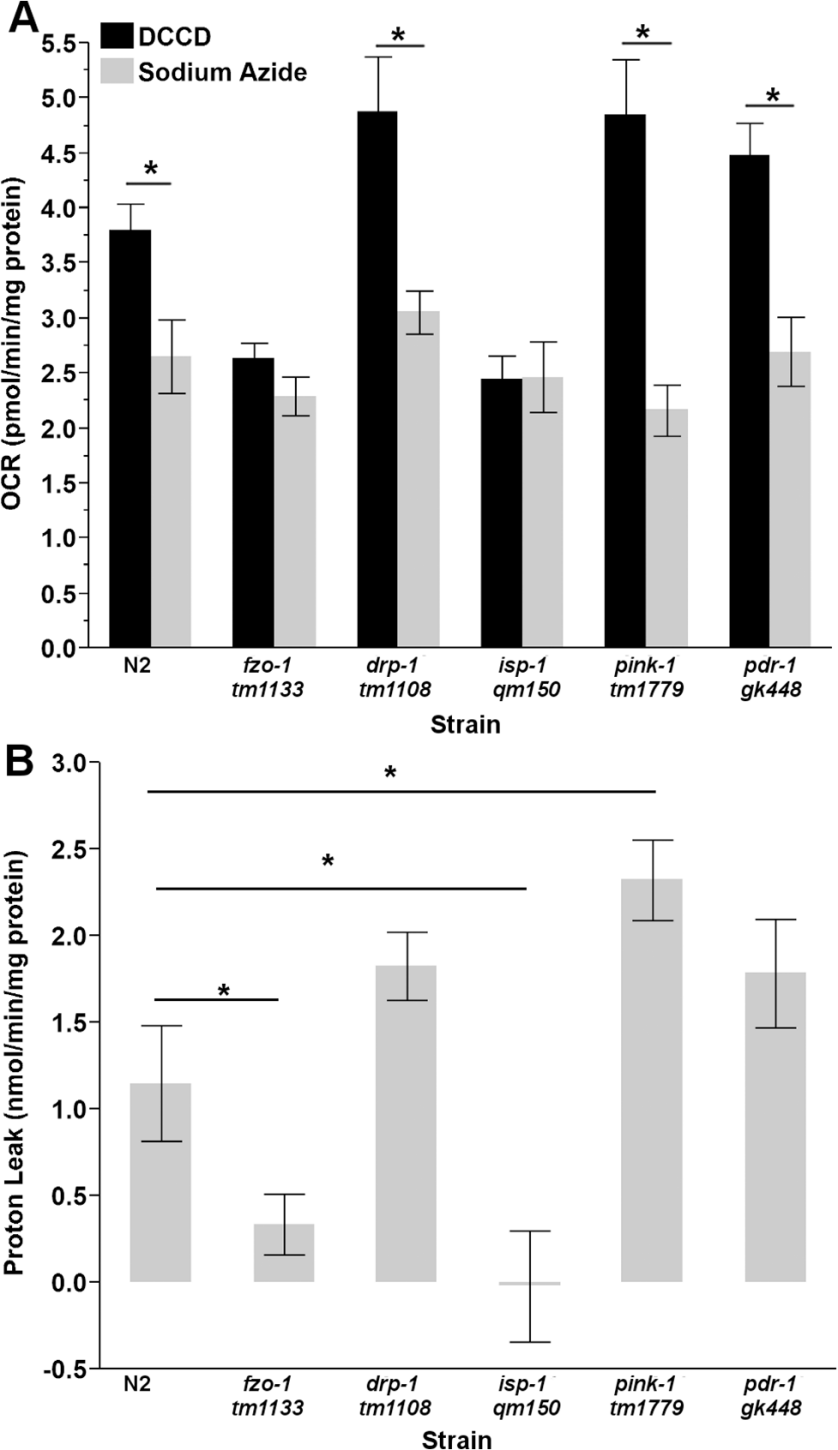
Proton leak in L4 nematodes. (A) Sodium azide and DCCD caused significantly different reductions in OCR in N2, *drp-1*, *pink-1*, and *pdr-1* nematodes (two way ANOVA, main effects of strain (P<0.0001), treatment (P<0.0001) and their interaction (p = 0.0002)), while *fzo-1* and *isp-1* responses were not significantly different. (B) L4 *fzo-1* and i*sp-1* nematodes have reduce proton leak, while *pink-1 C*. *elegans* have increased leak (one way ANOVA, P<0.0001). (n = 8–12). Asterisks (*) denote statistical significance. Bars ± SEM.

Basal OCR was significantly depressed upon injection of sodium azide in all strains tested (main effect of drug (P < .0001), but not strain (p = 0.15) or their interaction (p = 0.60)) (S17 Fig). Injection of DCCD and sodium azide caused significantly different reductions in OCR (two-way ANOVA, main effects of strain (P<0.0001), treatment (P<0.0001) and their interaction (P = 0.0002)) in N2, *pdr-1*, *pink-1* and *drp-1*, but not in *fzo-1* or *isp-1* (P = 0.017, P<0.0001, P<0.001, P<0.0001, P = 0.43, P = 0.96, respectively, for all pairwise comparisons) (Fig 5A). Comparing proton leak proved more challenging in nematodes than in cell culture, as we did not inject our ATP synthase inhibitor (DCCD) and complete respiratory inhibitor (sodium azide) into the same wells. Therefore, because we did not have matched samples to compare, we subtracted all azide response OCR measurements from the average DCCD measurement for each strain. Using this approach, we observed dramatic and significant strain differences (one way ANOVA, P<0.0001). Interestingly, *isp-1* and *fzo-1* nematodes had reduced proton leak compared to wild-type nematodes, while *pink-1* had significantly elevated proton leak (P = 0.005, P = 0.04, P = 0.004, respectively, for pairwise comparisons to control) (Fig 5B). Proton leak on a per worm basis is shown in S14 Fig; proton leak on a per nematode basis was not significantly different between strains (one way ANOVA, P = 0.051).

#### Mitochondrial Morphology

**Fig 6:**
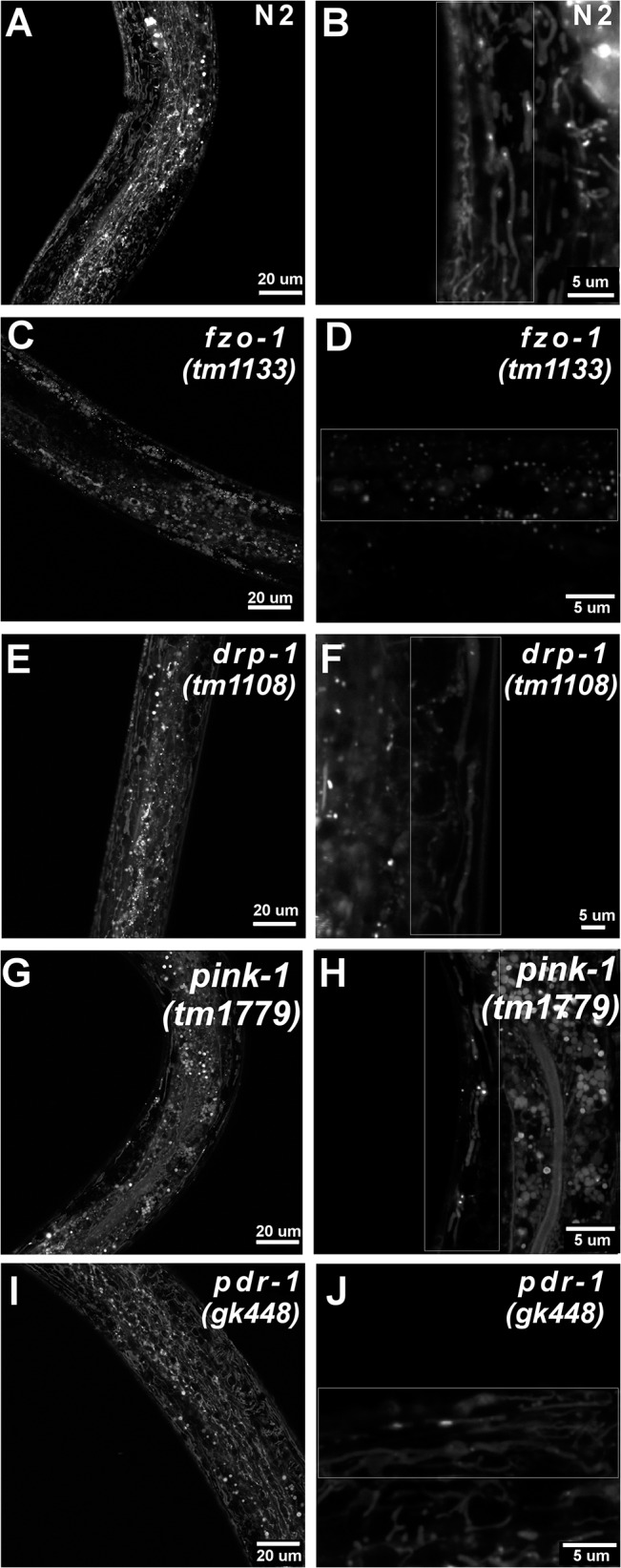
Mitochondrial morphology in wild-type and mutant *C*. *elegans*. Representative confocal images of mitochondrial morphology in wild-type (N2) (A-B) and mitochondrial mutant strains (*fzo-1* (C-D), *drp-1* (E-F), *pink-1* (G-H), *pdr-1* (I-J)) at L4. Left panel shows the sample at 63x, right panel shows a representative zoomed and cropped image from the z-stack (63x) used for image analysis.

As mitochondrial morphology can play a role in metabolic activity, we imaged muscle cell mitochondria and quantified the mitochondrial aspect ratio for each strain. The aspect ratio serves as a proxy for measuring the extent to which mitochondria are networked; a higher aspect ratio indicates more highly fused mitochondria (see Methods for more details). Results are summarized in Table 1 and representative images are shown in Fig 6. As expected, *fzo-1*-deficient nematodes had a significantly reduced aspect ratio compared to wild-type *C*. *elegans* (one way ANOVA, main effect of strain P<0.0001). Interestingly, both *pdr-1* and *pink-1* had significantly larger aspect ratios compared to N2 (one way ANOVA, main effect of strain P = 0.032 and P = 0.016, respectively). Surprisingly, *drp-1*-deficient nematode’s aspect ratio was not statistically different from wild-type *C*. *elegans* (one way ANOVA, P>0.05). Because this result was unexpected, *drp-1* nematodes were genotyped alongside N2 *C*. *elegans* and the 425bp deletion was confirmed. Although *isp-1*-deficient nematodes have been previously reported to have fragmented mitochondrial networks [64], we could not confirm this, as uptake of MitoTracker Red CMXROS dye was poor, likely due to *isp-1* nematode’s reduced mitochondrial membrane potential [59, 60].

## Discussion

Here we present a method to assess the fundamental parameters of mitochondrial function *in vivo*, using the model organism *C*. *elegans*: basal and maximal OCR, spare respiratory capacity, ATP-coupled respiration, and proton leak. Furthermore, we identify strain-specific differences in these parameters in mitochondrial fission (*drp-1*), fusion (*fzo-1*), mitophagy (*pdr-1* & *pink-1*), and ETC complex III (*isp-1*) deficient nematodes.

Although measuring mitochondrial function *in vivo* offers many advantages compared to *in vitro* assays, the method we describe here does have limitations. For example, the inability to inject drugs sequentially into each well reduces the throughput of the assay; however, this could potentially be overcome by adapting our protocol to the Seahorse XF^e^96 Analyzer. Furthermore, the dual probe nature of the XF^e^24 Analyzer allows for the simultaneous measure of both OCR and ECAR; however, ECAR measurements appear to have limited utility in *C*. *elegans*. Additionally, the Seahorse XF^e^24 Analyzer lacks cooling capacity, and tends to warm above room temperature as the assay progresses (on average to 25C). Thus, temperature may be a concern in longer assays. Storage of the XF^e^24 Analyzer in a temperature and humidity controlled enclosure may solve this issue.

Another limitation of this assay is the lack of a highly specific ATP synthase inhibitor that works in nematodes. DCCD, an F_O_F_1_ inhibitor, also inhibits other ATPases that can contribute to oxygen consumption [44], making ATP-linked respiration and proton leak measurements difficult to interpret. Unfortunately, the highly specific F_O_ inhibitor oligomycin appears to work only after very long incubation periods or in cuticle-deficient *bus-8* nematodes, limiting its practicality. Nevertheless, oligomycin could be used in conjunction with RNAi in a *bus-8*-deficient background to study the interactions between genetic knockdown, toxicant exposure and mitochondrial dysfunction. However, oligomycin only caused a 36% reduction in OCR, suggesting that only 36% of oxygen consumption is coupled to ATP production. This is a significantly lower value than normally reported, and we suspect that oligomycin is not completely inhibiting ATP synthase, even in a cuticle deficient background. A lack of complete inhibition could still be due to poor penetration of the nematode by oligomycin, or incomplete organismal diffusion leaving ATP synthase active in certain cell types. The most effective concentration of DCCD caused roughly a 20% greater decrease in OCR than oligomycin (S21 Fig); however, it seems unlikely that inhibition of nonspecific ATPases would contribute such a large percentage to OCR, further suggesting that oligomycin is not causing complete ATP synthase inhibition. We hypothesize that the 20% difference in DCCD and oligomycin responses are due to a combination of nonspecific inhibition of ATPases and incomplete inhibition of ATP synthase by oligomycin. Thus no matter which inhibitor is chosen for a study, results must be interpreted cautiously, and when possible confirmed via alternative methods, such as direct measurement of ATP levels.

Alterations in basal OCR were only observed in *drp-1* nematodes, which had an elevated OCR. In agreement with this finding, highly fused mitochondrial networks, which have been observed in *drp-1* (*tm1108*) knockout *C*. *elegans* [65], are often associated with increased metabolic activity [61]. Interestingly, we did not observe significantly different mitochondrial morphology between *drp-1* and N2 nematodes in the current study. Instead, wild-type nematodes appeared to have hyper-fused mitochondrial networks, which we speculate is either life-stage or cell-type specific (i.e. muscle cells). For example, at the L3/L4 transition a dramatic increase in mtDNA copy number occurs as demand for oxidative phosphorylation increases [35–37]. As highly fused mitochondria are typically more metabolically active [61] it is plausible that mitochondrial fusion also occurs in wild-type nematodes at the L3/L4 transition to help meet rising energy demands. However, it is important to note that mutations in *DRP1* are associated with human disease [18], and mitochondrial dysfunction has been reported in *drp-1* (*tm1108*) nematodes and Purkinje cells of DRP1 deficient mice [66]. Previously, we reported reduced ATP levels and an increased mtDNA:nDNA ratio in L4 *drp-1* nematodes [67], while others have reported decreased brood size [65].

Surprisingly, neither *fzo-1* nor *isp-1* nematodes had altered basal OCR, despite the fact that we (Fig 6) and others have observed fragmented mitochondrial networks in *fzo-1* and *isp-1* [41, 64, 68]. Reductions in basal OCR have been reported in *fzo-1* nematodes on a per worm basis [69], and mild reductions in L1 and mixed populations (normalized to either total protein or body volume) of *isp-1* nematodes [70, 71] have also been reported, neither of which were observed in our age-synchronized L4 populations. A limitation of these normalization methods is that they do not take into account potential strain specific differences in mitochondrial mass. The use of transgenic reporter strains expressing mitochondrial localized green fluorescent protein to estimate mitochondrial mass may improve normalization. However, similarly to *drp-1* deficient *C*. *elegans*, *isp-1* nematodes have also been reported to have reduced ATP levels [71, 72]**,** thus highlighting the necessity of measuring multiple endpoints in assessing mitochondrial health.

The fact that mutations in mitochondrial dynamics and complex III genes did not cause stronger phenotypes was surprising. One possibility is that this is the result of compensatory mechanisms, which have indeed been reported in ETC and mitochondrial dynamics mutant nematodes. For example, *fzo-1* nematodes appear to upregulate glycolysis in an attempt to maintain energy homeostasis [41], while *MFN1/MFN2-*deficient skeletal myocytes and Purkinje cells attempt, but fail, to maintain energy homeostasis by increasing mitochondrial biogenesis [73, 74]. Likewise, complex I (*gas-1*)-deficient nematodes have compensated for decreased complex I activity by upregulating transcription of other OXPHOS genes (in particular complex II and III), as well as genes involved in the TCA cycle, glycolysis and fatty acid metabolism [75]. Interestingly, complex III mutants (*isp-1*) appear to downregulate transcription of complex I, perhaps in an attempt to limit ETC-derived reactive oxygen species (ROS) [75]. Of course other, as yet unidentified, compensatory mechanisms are likely also playing a role, such that it is not surprising that major mitochondrial dysfunction was not detected until chemical challenge.

Exposure to the mitochondrial uncoupler FCCP revealed reduced maximal respiratory capacities in *isp-1*, *fzo-1*, and *drp-1* nematodes, while only *drp-1* and *fzo-1* had significantly reduced spare respiratory capacities. These results are somewhat surprising, as highly fused mitochondrial networks are often associated with increased metabolic activity [61]. However, mitochondrial homeostasis is maintained through the interlinked processes of fission and fusion, and a lack of either is associated with mitochondrial dysfunction in humans [16] and *C*. *elegans* [41]. Despite their shared loss in spare respiratory capacity, we have previously shown that *drp-1* nematodes are mildly resistant to UVC induced mtDNA damage, while *fzo-1 C*. *elegans* are hypersensitive [32, 67]. These differences are likely due to the buffering effect of increased fusion in *drp-1*, which has been lost in *fzo-1* nematodes. Nonetheless, these findings further highlight the importance of fission and fusion in maintaining proper mitochondrial function.

Interestingly, we observed increased proton leak in *pink-1*, and decreased leak in *fzo-1* and *isp-1* nematodes. Increased levels of ROS, the principle known inducer of uncoupling protein 2 (UCP2), which typically mediates inducible proton leak [62, 63], have been measured in *pink-1* deficient cell lines [76] and in mitochondria isolated from Parkinson’s disease patients [76–78]. In agreement with our findings, increased ROS production, decreased ATP levels and decreased membrane potential, suggestive of proton leak, were recently reported in *pink-1*-deficient nematodes [79]. However, the authors also measured increased basal OCR, and images of abnormal mitochondrial networks appeared fragmented, conflicting with our results [79]. Increased ROS production in *pink-1*-deficient nematodes could induce uncoupling activity, resulting in mild uncoupling, reduced ATP levels [67], and increased proton leak, which would limit further ETC-derived ROS. Uncoupling activity it not well understood in nematodes and the sole uncoupling protein homolog, a UCP4 homolog, does not appear to have uncoupling activity, but is instead a succinate transporter [80]. Interestingly, proton leak did not differ from wild-type levels in *pdr-1*-deficient nematodes, although *pdr-1*-deficient nematodes have recently been reported to have reduced ATP levels, reduced mitochondrial membrane potential, and increased basal OCR, suggestive of proton leak [79]. While *pink-1* and *pdr-1* both participate in the mitophagy pathway, different levels of proton leak in these two strains may be explained by the fact that *pink-1* is a kinase that has many functions in addition to mitophagy, including regulation of mitochondrial respiration and ROS production [81, 82]. Furthermore, emerging evidence suggests that cytosolic *pink-1* can promote cell survival and neuron differentiation [83–85]. Although proton leak differed between *pink-1* and *pdr-1*, we report here for the first time that both strains have mitochondrial networks that are highly fused, exhibiting significantly larger aspect ratios compared to wild-type nematodes, which is consistent with the strains’ shared loss of mitophagy, and thus reduced mitochondrial turnover.

Increased production of superoxide has been measured in *isp-1* nematodes by several groups [86, 87], which at first seems to conflict with our finding of decreased proton leak. However, superoxide appears to play both a beneficial and critical role in *isp-1’s* long lived phenotype, as supplementation with ROS scavengers reduced the long-lived phenotype of *isp-1* [87]. Instead of by increasing proton leak, *isp-1* nematodes may limit excessive ROS production by downregulating OXPHOS, including one of the main sites of ROS production, complex I [75]. Due to diminished complex I and III activity [75], it is likely that *isp-1* nematodes struggle to generate a proton gradient and proton leak would further limit ATP production in an organism that already has reduced ATP levels [71]. In agreement with this, several groups have reported reduced mitochondrial membrane potential in *isp-1*-deficient nematodes [59, 60], which also explains the lack of uptake of MitoTracker Red CMXROS dye, and our inability to quantify mitochondrial morphology in *isp-1*-deficient nematodes.

Mitochondrial function appears to deviate most from wild-type in *fzo-1* nematodes, which have reduced maximal respiratory capacity, spare respiratory capacity, proton leak, and a trend toward increased ATP-coupled oxygen consumption. These findings are not surprising as impaired respiration has been reported in *MFN1/MFN2* deficient cardiac myocytes [88], skeletal myocytes [74], and in Purkinje cells [73]. In accord with mitochondrial dysfunction, *fzo-1* nematodes have highly fragmented mitochondria and develop slowly; however, their AMP/ATP ratio does not appear altered, suggesting that loss of *fzo-1* does not significantly alter energy production [41]. Instead, *fzo-1*-deficient nematodes may upregulate glycolysis in order to maintain energy homeostasis [41] and/or alter behavior to reduce ATP use. In agreement with our findings, decreased proton leak has been reported in 10T1/2 cells transfected with antisense mouse *MFN2* [89]; however, this group also reported reductions in basal OCR. Interestingly, upregulation of *ANT3*, but not the ROS inducible *UCP2*, has been observed in mitochondria isolated from CMT2A patients [90], suggestive of increased proton leak, but not superoxide production, which conflicts with our findings. However, to our knowledge, mitochondrial uncoupling has not been investigated in *fzo-1* nematodes, and as superoxide production does not exceed wild-type levels, mitochondrial uncoupling would not be expected to be upregulated via ROS production [69]. Since proton leak would further reduce OXPHOS derived ATP production by dissipating the proton gradient, it is likely that proton leak would not be advantageous to *fzo-1* nematodes.

Reports of ATP-coupled respiration vary in *in vitro* studies of *MFN2*-deficiency. Mitochondria in fibroblasts isolated from CMT2A patients have been reported to have decreased coupling [90, 91], while others have reported no effect [89, 92]. Interestingly, *MFN2* knockout L6E9 mytotubes appears to repress OXPHOS, as nuclear encoded subunits of complex I, II, III, and V are all downregulated, while glucose transport, and lactate production were upregulated, suggesting a compensatory switch to glycolysis to maintain ATP levels [92]. On the other hand, fibroblasts from CMT2A patients, which have reduced coupling efficiency, maintained ATP levels by increasing oxygen consumption and complex II activity [90]. Although not significant, we did observe a trend toward increased ATP-coupled respiration in *fzo-1* nematodes (one way ANOVA, P = 0.07, S11B Fig), which is in agreement with our finding of decreased proton leak, as leak tends to result in mild uncoupling. It is likely that many of these discrepancies are due to the complex nature of *MFN2*, the role it plays in different cell types, or the varying effects knockout versus knockdown versus mutations ultimately have on protein function.

## Conclusions

We describe measurement of the fundamental parameters of mitochondrial function in the model organism *C*. *elegans*, and demonstrate differences in these parameters in nematodes carrying mutations in genes coding for proteins involved in fission, fusion, mitophagy, and the ETC that result in altered mitochondrial morphology. Interestingly, many strain differences were not apparent until chemical challenge, highlighting the importance of carrying out tests that incorporate both genetic differences and toxicant exposure before definitive conclusions can be drawn about overall mitochondrial function and resilience.

## Supporting Information

S1 Fig Response of L4 N2 nematodes to sodium azide alone and post-FCCP. Response to sodium azide was assessed statistically with a one way ANOVA (P = 0.0005). Asterisks (*) denote statistical significance. Bars ± SEM.
(TIFF)

S2 Fig Basal extracellular acidification rate (ECAR) in L4 N2 and *fzo-1* nematodes. One way ANOVA (P>0.05). (n = 11). Bars ± SEM.
(TIFF)

S3 Fig Oligomycin does not reduce OCR in L4 N2 nematodes. Representative Seahorse output data. (n = 4 for each concentration shown).
(TIFF)

S4 Fig Titration of Dicyclohexylcarbodiimide in L4 N2 nematodes. Significance assessed with a one way ANOVA (P<0.0001), followed by student’s T-tests for pairwise comparisons. Asterisks (*) denote statistical significance. Bars ± SEM.
(TIFF)

S5 Fig Effect of DMSO concentration of efficacy of 20μM DCCD. Significance assessed with a one way ANOVA (P<0.0001), followed by student’s T-tests for pairwise comparisons. Asterisks (*) denote statistical significance. Bars ± SEM.
(TIFF)

S6 Fig 2,4-Dinitrophenol fails to increase OCR in L4 N2 nematodes. Representative Seahorse XF^e^24 output data.
(TIFF)

S7 Fig Titration of FCCP in L4 N2 nematodes. Significance assessed with a one way ANOVA (main effect of treatment, P<0.0001). Asterisks (*) denote statistical significance. Bars ± SEM.
(TIFF)

S8 Fig Effect of DMSO concentration of efficacy of 15μM FCCP. Representative Seahorse XF^e^24 output data. (n = 4 for each concentration shown).
(TIFF)

S9 Fig Titration of sodium azide in L4 N2 nematodes. Significance assessed with a one way ANOVA (main effect of treatment, P<0.0001). Asterisks (*) denote statistical significance. Bars ± SEM.
(TIFF)

S10 Fig Basal OCR is elevated in *drp-1* and reduced in *isp-1* L4 *C*. *elegans* on a per nematode basis. Asterisks (*) denote statistical significance. Bars ± SEM.
(TIFF)

S11 Fig ATP coupled respiration. (A) 20μM DCCD caused a significant reduction in OCR in all strains (two way ANOVA, main effects of strain and treatment, P<0.0001 for both, but not their interaction). (B) A trend in increased ATP coupled respiration was observed in *fzo-1* nematodes (one way ANOVA, P = 0.07). (n = 12–16). Asterisks (*) denote statistical significance. Bars ± SEM.
(TIFF)

S12 Fig ATP coupled respiration per nematode. (A) 20μM DCCD caused a significant reduction in OCR in all strains (two way ANOVA, main effects of strain and treatment, P<0.0001 for both, but not their interaction), (B) but no significant effect on ATP-linked respiration was observed. Asterisks (*) denote statistical significance. Bars ± SEM.
(TIFF)

S13 Fig Maximal and spare respiratory capacity in L4 *C*. *elegans* on a per nematode basis. (A) *fzo-1*, *isp-1*, and *drp-1* nematodes have a significantly reduced FCCP response (two way ANOVA, main effects of strain, treatment, and their interaction, P = 0.0001 for all) (A) and (B) spare respiratory capacity. Asterisks (*) denote statistical significance. Bars ± SEM.
(TIFF)

S14 Fig Proton leak per L4 nematode. (A) Effect of sodium azide and DCCD on OCR (two way ANOVA, P>0.05) and (B) proton leak per nematode measured (two way ANOVA, P>0.05).
(TIFF)

S15 Fig Effect of 2% DMSO on OCR normalized to total protein. DMSO had no effect on OCR in any of the strains tested (two way ANOVA, P>0.05). Bars ± SEM.
(TIFF)

S16 Fig Proton leak per L4 nematode. DMSO had no effect on OCR in any of the strains tested (two way ANOVA, P>0.05). Bars ± SEM.
(TIFF)

S17 Fig Basal OCR and sodium azide response. Sodium azide caused a significant reduction in OCR in all strains tested (one was ANOVA, P<0.0001). Bars ± SEM.
(TIFF)

S18 Fig Oligomycin pre-incubation with *bus-8*-deficient nematodes. A 12 hour pre-incubation with oligomycin caused a significant reduction in OCR (one way ANOVA, P<0.0001). Bars ± SEM.
(TIFF)

S19 Fig Oligomycin titration with *bus-8*-deficient nematodes. Treatment with oligomycin caused a significant reduction in OCR (one way ANOVA, P = 0.0007). Bars ± SEM.
(TIFF)

S20 Fig DCCD titration with *bus-8*-deficient nematodes. Treatment with DCCD caused a significant reduction in OCR (one way ANOVA, P<0.0001). Bars ± SEM.
(TIFF)

S21 Fig Effect of 20μM DCCD and 50μM oligomycin in *bus-8* nematodes. 20μM DCCD caused a significantly greater reduction in OCR than 50μM oligomycin in *bus-8* nematodes (one way ANOVA, main effect of treatment P = 0.0005). Bars ± SEM.
(TIFF)

S1 File Contains all data used in the preparation of in text figures and Supporting Information figures. (XLSX)
