# Correction: Mitochondrial Morphology and Fundamental Parameters of the Mitochondrial Respiratory Chain Are Altered in *Caenorhabditis elegans* Strains Deficient in Mitochondrial Dynamics and Homeostasis Processes

**DOI:** 10.1371/journal.pone.0168738

**Published:** 2016-12-15

**Authors:** Anthony L. Luz, John P. Rooney, Laura L. Kubik, Claudia P. Gonzalez, Dong Hoon Song, Joel N. Meyer

In Figs 2, 3, 4 and 5, and S1, S4, S5, S7, S9, S10, S11, S12, S13, S14, S16, S17, S18, S19 and S20 Figs, the y-axes are incorrectly labeled “pmol/min/mg protein”, when they should be labeled “nmol/min/mg protein”. Please see the corrected files here.

**Fig 2:**
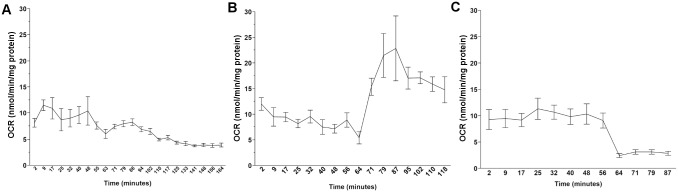
Representative Seahorse XFe24 output data for L4 N2 nematodes dosed with (A) DCCD, (B) FCCP, and (C) sodium azide.

**Fig 3:**
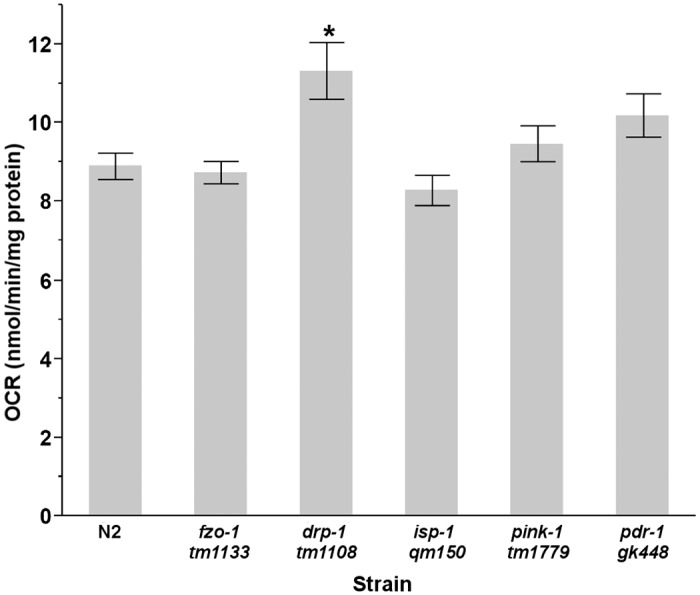
Basal OCR is elevated in L4 *drp-1* nematodes. Statistical significance was analyzed via a one way ANOVA (main effect of strain, P<0.0001) (n = 31–45). Asterisks (*) denote statistical significance. Bars ± SEM.

**Fig 4:**
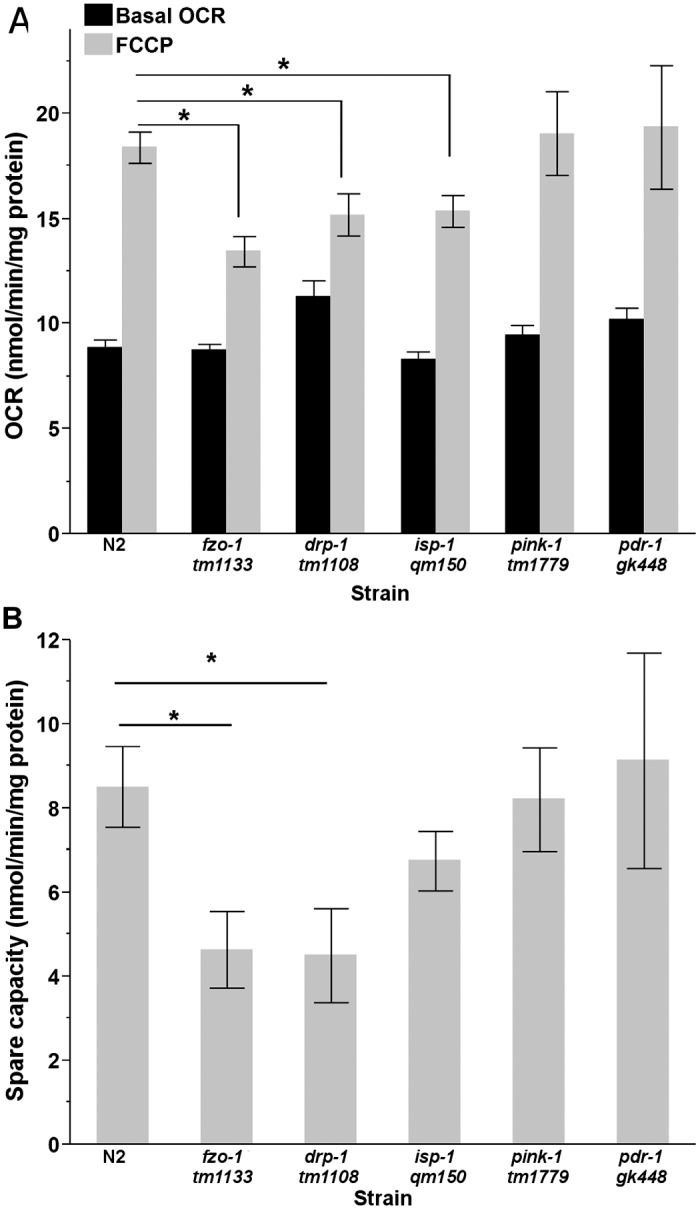
Maximal and spare respiratory capacity in L4 nematodes. (A) Treatment with FCCP caused a significant increase in OCR in all strains (two way ANOVA, main effects of strain, treatment and their interaction, P<0.0001 for all); however, L4 *fzo-1*, *drp-1* and *isp-1* had reduced maximal respiratory capacity compared to N2 nematodes (Student’s t-test, p = 0.03, p<0.0001, p = 0.01, respectively). (B) Spare respiratory capacity was reduced in *fzo-1* and *drp-1*, compared to wild-type nematodes (one way ANOVA, P = 0.022). (n = 12–20). Asterisks (*) denote statistical significance. Bars ± SEM.

**Fig 5:**
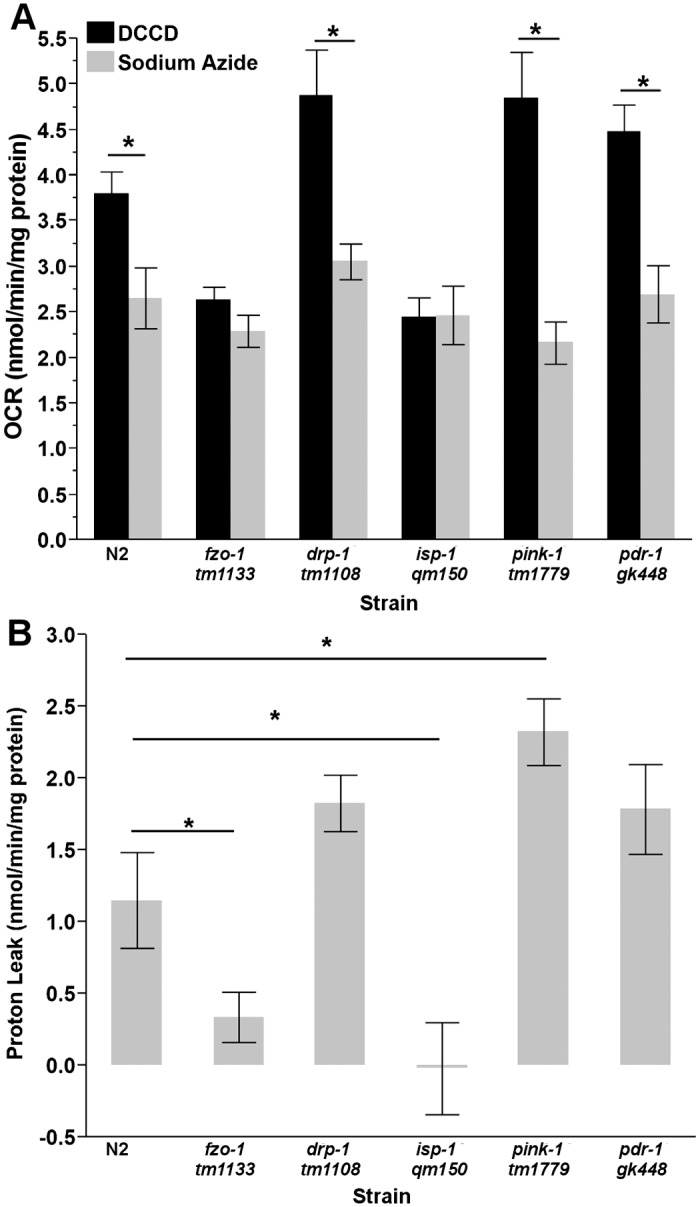
Proton leak in L4 nematodes. (A) Sodium azide and DCCD caused significantly different reductions in OCR in N2, *drp-1*, *pink-1*, and *pdr-1* nematodes (two way ANOVA, main effects of strain (P<0.0001), treatment (P<0.0001) and their interaction (p = 0.0002)), while *fzo-1* and *isp-1* responses were not significantly different. (B) L4 *fzo-1* and *isp-1* nematodes have reduce proton leak, while *pink-1 C*. *elegans* have increased leak (one way ANOVA, P<0.0001). (n = 8–12). Asterisks (*) denote statistical significance. Bars ± SEM.

## Supporting Information

S1 Fig Response of L4 N2 nematodes to sodium azide alone and post-FCCP. Response to sodium azide was assessed statistically with a one way ANOVA (P = 0.0005). Asterisks (*) denote statistical significance. Bars ± SEM.
(TIFF)

S4 Fig Titration of Dicyclohexylcarbodiimide in L4 N2 nematodes. Significance assessed with a one way ANOVA (P<0.0001), followed by student’s T-tests for pairwise comparisons. Asterisks (*) denote statistical significance. Bars ± SEM.
(TIFF)

S5 Fig Effect of DMSO concentration of efficacy of 20μM DCCD. Significance assessed with a one way ANOVA (P<0.0001), followed by student’s T-tests for pairwise comparisons. Asterisks (*) denote statistical significance. Bars ± SEM.
(TIFF)

S7 Fig Titration of FCCP in L4 N2 nematodes. Significance assessed with a one way ANOVA (main effect of treatment, P<0.0001). Asterisks (*) denote statistical significance. Bars ± SEM.
(TIFF)

S9 Fig Titration of sodium azide in L4 N2 nematodes. Significance assessed with a one way ANOVA (main effect of treatment, P<0.0001). Asterisks (*) denote statistical significance. Bars ± SEM.
(TIFF)

S10 Fig Basal OCR is elevated in drp-1 and reduced in isp-1 L4 C. *elegans* on a per nematode basis. Asterisks (*) denote statistical significance. Bars ± SEM.
(TIFF)

S11 Fig ATP coupled respiration. (A) 20μM DCCD caused a significant reduction in OCR in all strains (two way ANOVA, main effects of strain and treatment, P<0.0001 for both, but not their interaction). (B) A trend in increased ATP coupled respiration was observed in fzo-1 nematodes (one way ANOVA, P = 0.07). (n = 12–16). Asterisks (*) denote statistical significance. Bars ± SEM.
(TIFF)

S12 Fig ATP coupled respiration per nematode. (A) 20μM DCCD caused a significant reduction in OCR in all strains (two way ANOVA, main effects of strain and treatment, P<0.0001 for both, but not their interaction), (B) but no significant effect on ATP-linked respiration was observed. Asterisks (*) denote statistical significance. Bars ± SEM.
(TIFF)

S13 Fig Maximal and spare respiratory capacity in L4 C. elegans on a per nematode basis. (A) *fzo-1*, *isp-1*, and *drp-1* nematodes have a significantly reduced FCCP response (two way ANOVA, main effects of strain, treatment, and their interaction, P = 0.0001 for all) (A) and (B) spare respiratory capacity. Asterisks (*) denote statistical significance. Bars ± SEM.
(TIFF)

S14 Fig Proton leak per L4 nematode. (A) Effect of sodium azide and DCCD on OCR (two way ANOVA, P>0.05) and (B) proton leak per nematode measured (two way ANOVA, P>0.05).
(TIFF)

S16 Fig Proton leak per L4 nematode. DMSO had no effect on OCR in any of the strains tested (two way ANOVA, P>0.05). Bars ± SEM.
(TIFF)

S17 Fig Basal OCR and sodium azide response. Sodium azide caused a significant reduction in OCR in all strains tested (one was ANOVA, P<0.0001). Bars ± SEM.
(TIFF)

S18 Fig Oligomycin pre-incubation with *bus-8-*deficient nematodes. A 12 hour pre-incubation with oligomycin caused a significant reduction in OCR (one way ANOVA, P<0.0001). Bars ± SEM.
(TIFF)

S19 Fig Oligomycin titration with *bus-8-*deficient nematodes. Treatment with oligomycin caused a significant reduction in OCR (one way ANOVA, P = 0.0007). Bars ± SEM.
(TIFF)

S20 Fig DCCD titration with *bus-8-*deficient nematodes. Treatment with DCCD caused a significant reduction in OCR (one way ANOVA, P<0.0001). Bars ± SEM.
(TIFF)
