# Supplementary figures and images for: Mitochondrial Morphology and Fundamental Parameters of the Mitochondrial Respiratory Chain Are Altered in Caenorhabditis elegans Strains Deficient in Mitochondrial Dynamics and Homeostasis Processes
Source: PLoS One. 2015 Jun 24;10(6):e0130940. doi: 10.1371/journal.pone.0130940 (PMC4480853; doi:10.1371/journal.pone.0130940)

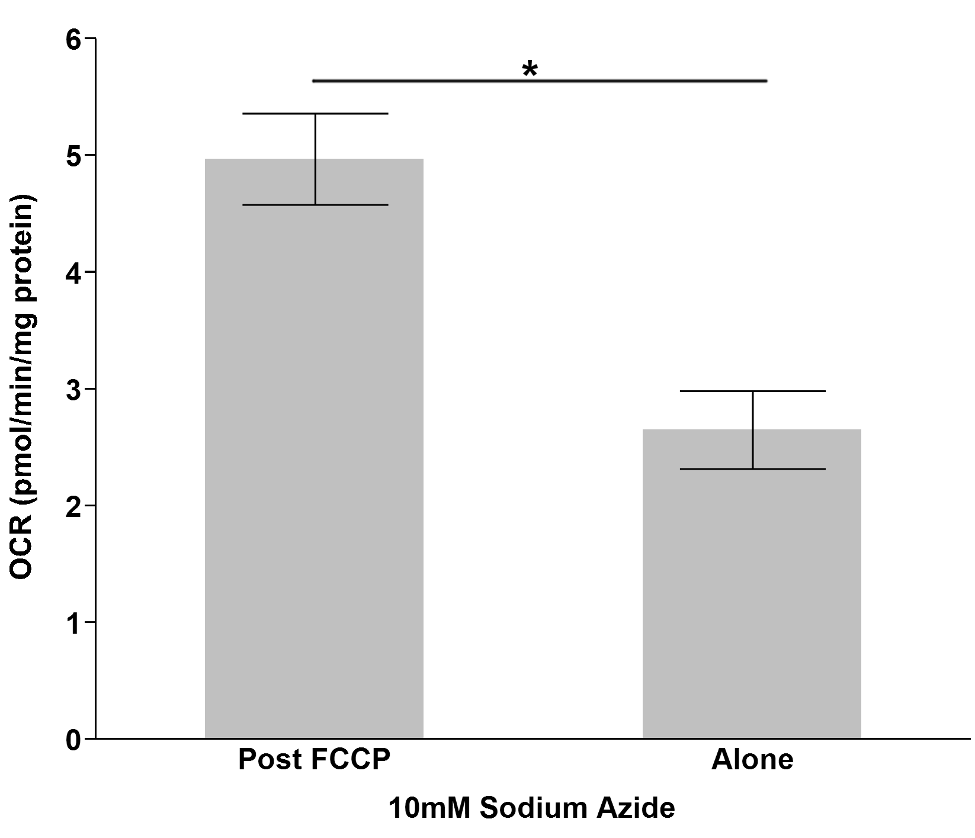

Supplement: S1 Fig — Response to sodium azide was assessed statistically with a one way ANOVA (P = 0.0005). Asterisks (*) denote statistical significance. Bars ± SEM. (TIFF) [file pone.0130940.s001.tiff]

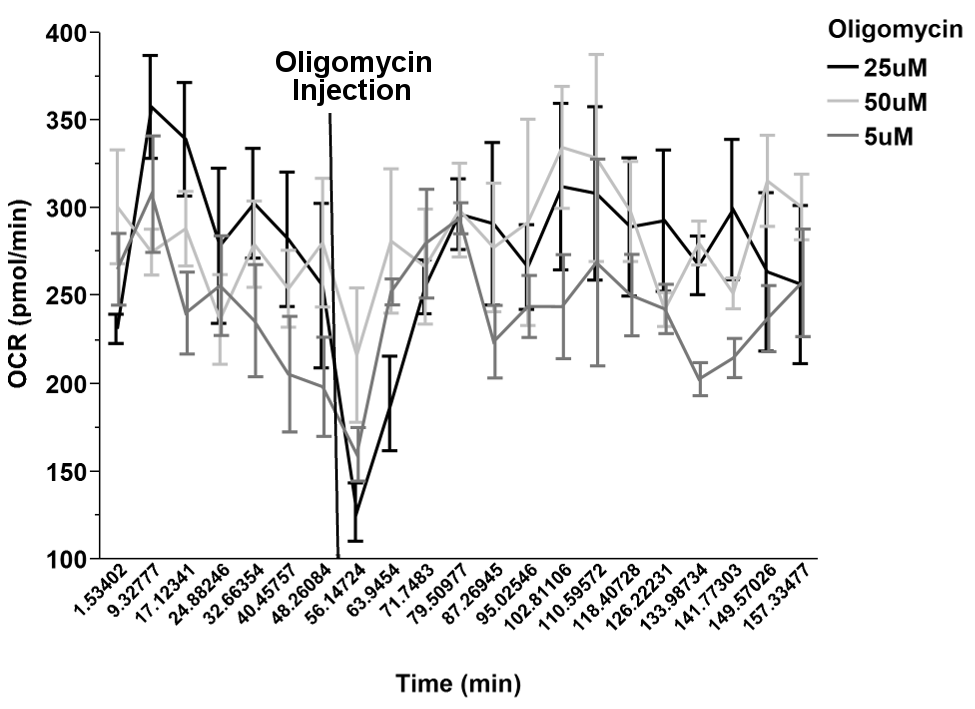

Supplement: S3 Fig — Representative Seahorse output data. (n = 4 for each concentration shown). (TIFF) [file pone.0130940.s003.tiff]

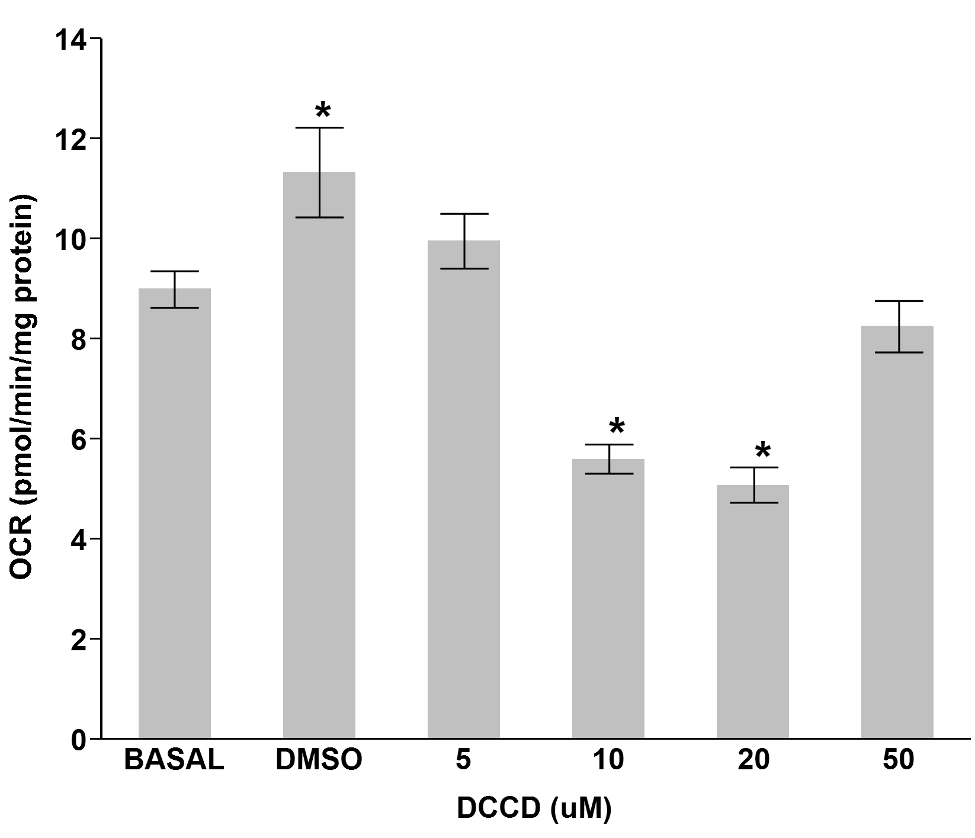

Supplement: S4 Fig — Significance assessed with a one way ANOVA (P<0.0001), followed by student’s T-tests for pairwise comparisons. Asterisks (*) denote statistical significance. Bars ± SEM. (TIFF) [file pone.0130940.s004.tiff]

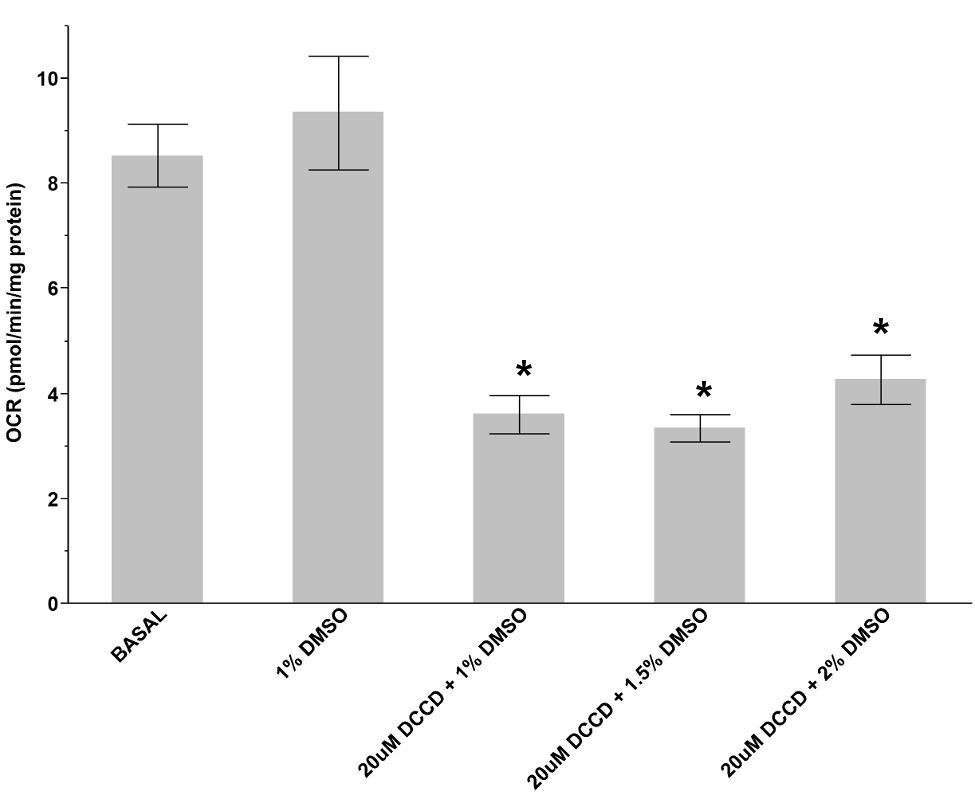

Supplement: S5 Fig — Significance assessed with a one way ANOVA (P<0.0001), followed by student’s T-tests for pairwise comparisons. Asterisks (*) denote statistical significance. Bars ± SEM. (TIFF) [file pone.0130940.s005.tiff]

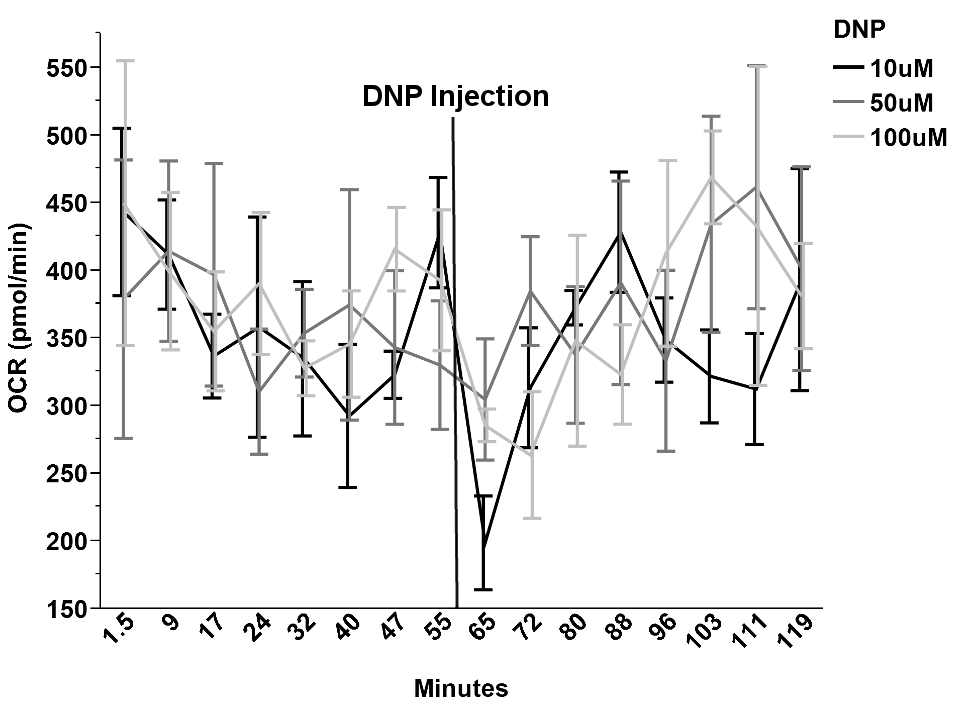

Supplement: S6 Fig — Representative Seahorse XFe24 output data. (TIFF) [file pone.0130940.s006.tiff]

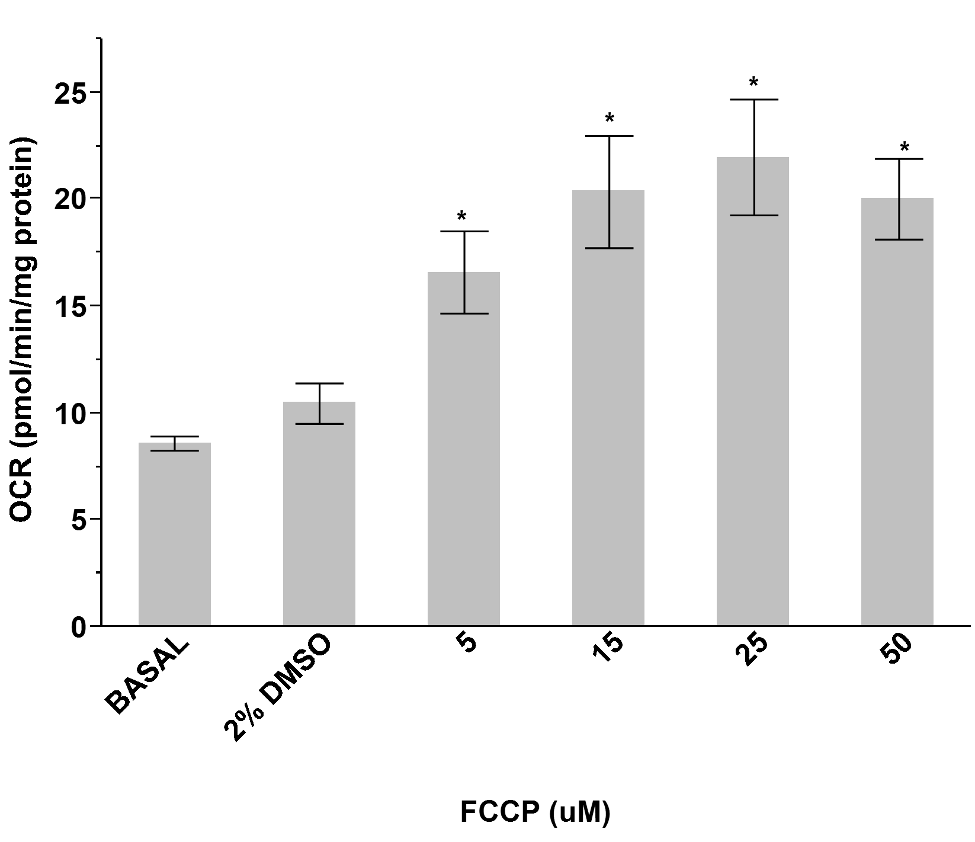

Supplement: S7 Fig — Significance assessed with a one way ANOVA (main effect of treatment, P<0.0001). Asterisks (*) denote statistical significance. Bars ± SEM. (TIFF) [file pone.0130940.s007.tiff]

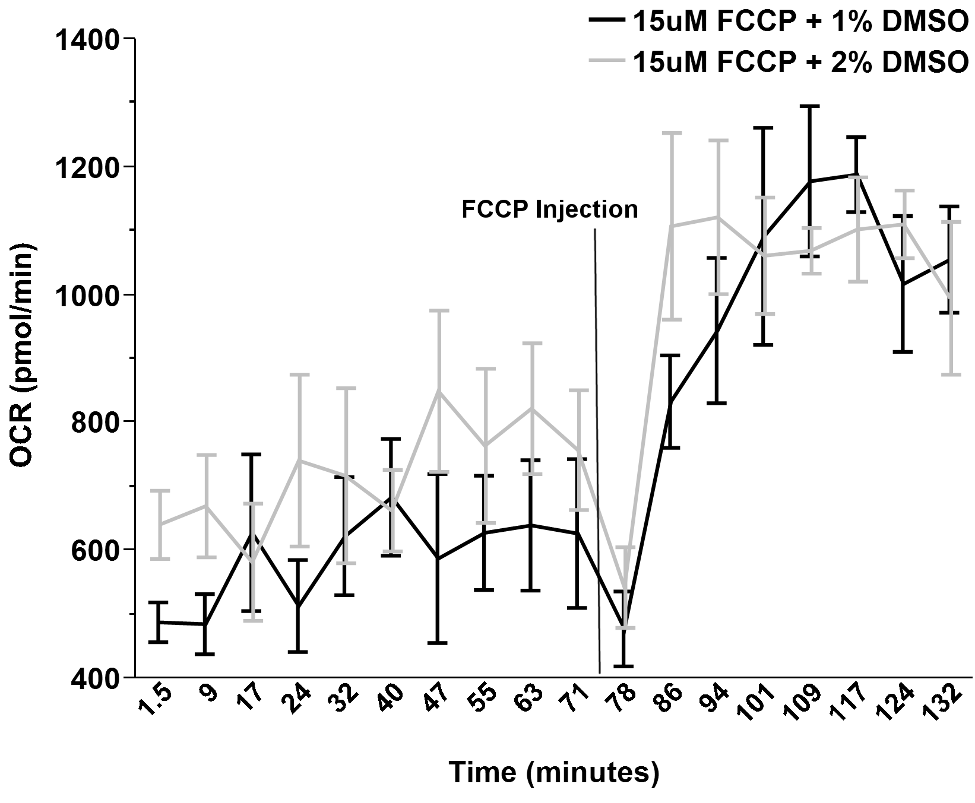

Supplement: S8 Fig — Representative Seahorse XFe24 output data. (n = 4 for each concentration shown). (TIFF) [file pone.0130940.s008.tiff]

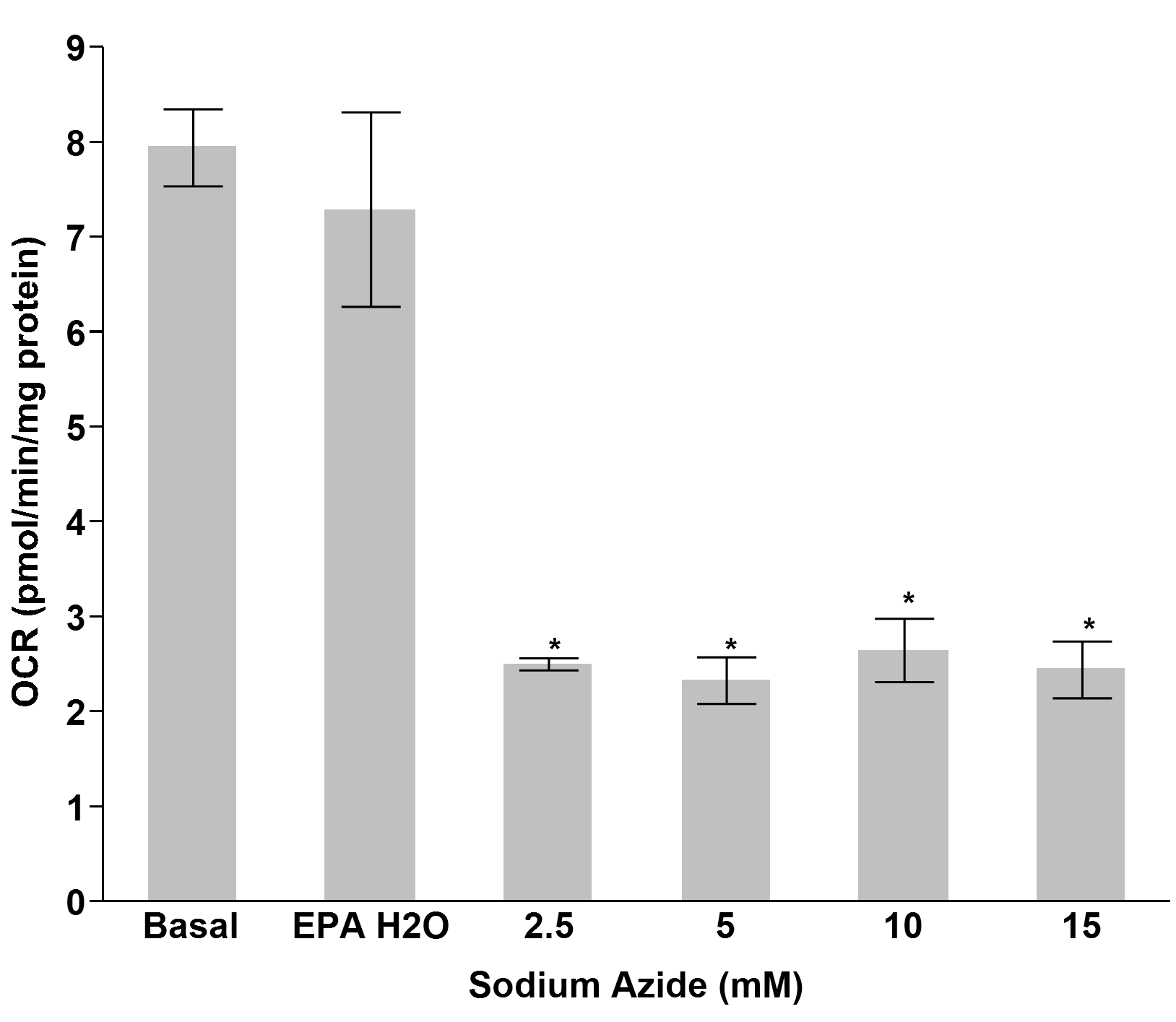

Supplement: S9 Fig — Significance assessed with a one way ANOVA (main effect of treatment, P<0.0001). Asterisks (*) denote statistical significance. Bars ± SEM. (TIFF) [file pone.0130940.s009.tiff]
