# Supplementary figures and images for: Correction: Mitochondrial Morphology and Fundamental Parameters of the Mitochondrial Respiratory Chain Are Altered in Caenorhabditis elegans Strains Deficient in Mitochondrial Dynamics and Homeostasis Processes
Source: PLoS One. 2016 Dec 15;11(12):e0168738. doi: 10.1371/journal.pone.0168738 (PMC5158073; doi:10.1371/journal.pone.0168738)

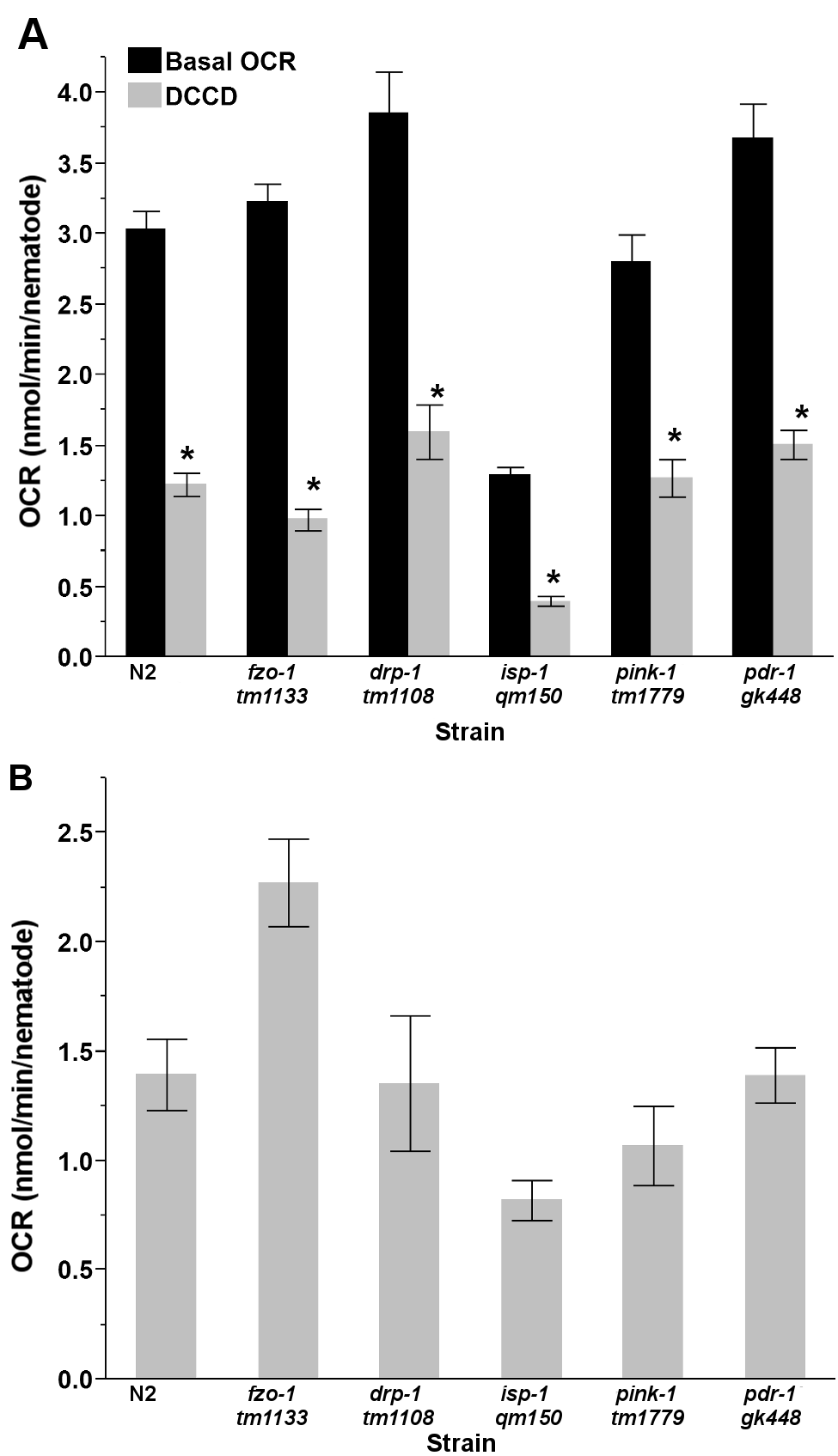

Supplement: S12 Fig — (A) 20μM DCCD caused a significant reduction in OCR in all strains (two way ANOVA, main effects of strain and treatment, P<0.0001 for both, but not their interaction), (B) but no significant effect on ATP-linked respiration was observed. Asterisks (*) denote statistical significance. Bars ± SEM. (TIFF) [file pone.0168738.s008.tiff]
